# Development of a TGFβ—IL-2/15 Switch Receptor for Use in Adoptive Cell Therapy

**DOI:** 10.3390/biomedicines11020459

**Published:** 2023-02-04

**Authors:** Carole Beck, Nicholas Paul Casey, Irene Persiconi, Neda Nejati Moharrami, Adam Sike, Yixin Jin, Jon Amund Kyte

**Affiliations:** 1 Department of Cancer Immunology, Institute for Cancer Research, Oslo University Hospital, 0424 Oslo, Norway; 2 Department of Clinical Cancer Research, Oslo University Hospital, 0424 Oslo, Norway

**Keywords:** chimeric antigen receptor, immune suppression, tumor microenvironment, transforming growth factor β, prostate cancer, switch receptor

## Abstract

Therapy employing T cells modified with chimeric antigen receptors (CARs) is effective in hematological malignancies but not yet in solid cancers. CAR T cell activity in solid tumors is limited by immunosuppressive factors, including transforming growth factor β (TGFβ). Here, we describe the development of a switch receptor (SwR), in which the extracellular domains of the TGFβ receptor are fused to the intracellular domains from the IL-2/15 receptor. We evaluated the SwR in tandem with two variants of a CAR that we have developed against STEAP1, a protein highly expressed in prostate cancer. The SwR-CAR T cell activity was assessed against a panel of STEAP1^+/−^ prostate cancer cell lines with or without over-expression of TGFβ, or with added TGFβ, by use of flow cytometry cytokine and killing assays, Luminex cytokine profiling, cell counts, and flow cytometry phenotyping. The results showed that the SwR-CAR constructs improved the functionality of CAR T cells in TGFβ-rich environments, as measured by T cell proliferation and survival, cytokine response, and cytotoxicity. In assays with four repeated target-cell stimulations, the SwR-CAR T cells developed an activated effector memory phenotype with retained STEAP1-specific activity. In conclusion, the SwR confers CAR T cells with potent and durable in vitro functionality in TGFβ-rich environments. The SwR may be used as an add-on construct for CAR T cells or other forms of adoptive cell therapy.

## 1. Introduction

Chimeric antigen receptors (CARs) are employed for retargeting T cells against tumor-associated antigens. CAR T cell therapy shows clinical efficacy against hematological cancers [1] but not yet in solid tumors, except for individual cases [2]. The limited effect in solid cancers has, in part, been attributed to an immunosuppressive tumor microenvironment [2,3,4]. TGFβ is a key mediator of immunosuppression across most solid cancers [5,6,7,8,9]. Here, we reported the development of a TGFβ-switch receptor (TGFβ-SwR) for use in CAR T cells and in other adoptive cell therapy approaches. The receptor is intended to make the T cells resistant to TGFβ-mediated immune suppression and, moreover, to turn the TGFβ- binding into a stimulatory signal for increased CAR T expansion and functionality.

TGFβ inhibits T cell activity through binding to the receptors TGFβRI and TGFβRII. The binding induces hetero-dimerization of the receptors and phosphorylation of the intracellular signal mediators SMAD2 and SMAD3, which, in turn, induce a suppressive transcriptional program [9]. This leads to an attenuated T cell response, with reduced T cell proliferation, cytokine production, and cytotoxicity [10,11]. Further, TGF-β drives T cell differentiation into regulatory T cells [12]. Several CAR T approaches for countering TGFβ-mediated immunosuppression are being investigated [13]. This includes a receptor that binds TGFβ by an scFv-binding domain and stimulates the engineered T cell through CD28-signaling [14] and a hybrid receptor fusing the TGFβ receptor II exodomain with the endodomain of 4-1BB [15]. The first approach to be brought into clinical testing is a double negative, non-signaling TGFβ-receptor (dnTGFβ-R) [16], which has been evaluated in patients with castration-resistant metastatic prostate cancer [17]. The study showed acceptable safety and a PSA30 response in 4 of 10 patients [18]. 

In the switch receptor described herein, we employ the endodomain of the shared β chain between the receptors for IL-2 and IL-15 (IL-2/15 Rβ). This domain has, to our knowledge, not previously been explored for this purpose. IL-2 and IL-15 share two out of three receptor units but have only partially overlapping functions. IL-2 promotes T cell proliferation, initial effector differentiation, and cytokine production, but also activation-induced cell death, while IL-15 is important for the memory development and survival of CD8^+^ cytotoxic T cells [19,20]. The intracellular signaling pathways downstream of IL-2/IL-15 stimulation are largely similar, with phosphorylation and activation of the JAK kinases, followed by recruitment and phosphorylation of STAT transcription factors [19,20].

TGFβ is secreted at high levels in prostate cancer [17], which is the most common cancer among males and a leading cause of cancer-related death [21]. We have developed a CAR targeting the 339–amino acid cell surface protein Six-Transmembrane Epithelial Antigen of Prostate-1 (STEAP1) [22]. STEAP1 is expressed in ~90% of prostate cancers and in subgroups of other cancers, such as Ewing sarcoma, lung cancer, bladder cancer, breast cancer, pancreatic cancer, glioblastoma, ovarian cancer, leukemia, lymphoma, and head and neck cancer [23,24,25,26]. Across multiple cancer forms, STEAP1 has been associated with tumor proliferation, progression, and invasiveness [23,24,27], including tumor invasion into the peritoneum [28,29,30,31,32]. Among normal tissues, STEAP1 is mainly expressed in the prostate, which is not a vital organ. The first published trial with a STEAP1 targeting antibody–drug-conjugate indicated a favorable safety profile, with no STEAP1-directed toxicity [33].

As described below, we have evaluated the TGFβ-switch receptor in tandem with the STEAP1 CAR. We recently reported that this STEAP1 CAR confers recipient T cells with potent target-specific functionality, including the production of multiple cytokines and the capacity to kill cancer cells [22]. In both subcutaneous and metastatic xenograft mouse models of prostate cancer, the STEAP1 CAR T cells infiltrated tumors, significantly inhibited tumor growth, and extended survival [22]. Below, we describe the development of the TGFβ–IL-2/15 Rβγ switch receptor and characterize the functionality of single constructs encoding the TGFβ-SwR in tandem with two variants of the STEAP1 CAR.

## 2. Methods

### 2.1. Cell lines, Primary T Cell Culture, and Activation

The prostate cancer cell lines LNCaP (American Type Culture Collection [ATCC], CRL-1740), 22Rv1 (ATCC, CRL-2505), C4-2B (a generous gift from Leland Chung; Cedars-Sinai Medical Center, CA), PC3 (ATCC, CRL-1435) and DU145 (ATCC, HTB-81), and the NALM-6 leukemia cell line (ATCC, CRL 3273) were cultured as previously described [22]. The Phoenix-AMPHO cell line (ATCC, CRL-3213) was maintained in DMEM 4.5 g/L glucose, supplemented with 10% Hyclone FBS (Sigma Aldrich, Oslo, Norway) and 100 U/mL penicillin/streptomycin. Peripheral blood mononuclear cells (PBMCs) were isolated from healthy donor buffy coats using Lymphoprep (Axis-Shield, Oslo, Norway) and cultured in RPMI with 10% heat-inactivated FBS and 100 U/mL penicillin/streptomycin. T cells from PBMCs were activated for 2 days with 1 µg/mL anti-CD3 (clone OKT3, Biolegend, Oslo, Norway, 317347) and 1 µg/mL anti-CD28 (clone CD28.6, Thermo Fisher Scientific, Oslo, Norway, 16-0288-81), and cultured in the presence of 100 IU/mL rhIL-2 (R&D Systems, Abingdon, UK, 202-IL).

### 2.2. Generation of 22Rv1-KO and 22Rv1-TGFβ Cell Lines

The STEAP1 gene was inactivated in the 22Rv1 cell line using a CRISPR/Cas9-mediated gene editing approach, as previously described [22]. A 22Rv1 cell line overexpressing TGFβ was generated using the pHAGE-TGFB1 (a gift from Gordon Mills and Kenneth Scott, Houston, TX, USA; Addgene, #116799) lentiviral vector. Lentiviral particles were produced as previously described [22]. The 22Rv1 cells were transduced for 48 h in the presence of 4 µg/mL of polybrene, and expanded. TGFβ was co-expressed with the marker gene EGFP. Cells over-expressing TGFβ (GFP^+^) were sorted to 98% purity using a Sony SH800 cell sorter (Sony, Weybridge, UK).

### 2.3. CAR Design

The JK11 STEAP1 CAR and the CD19 CAR were constructed as described previously [22]. The dominant-negative mutant TGFβ receptor—STEAP1 CAR (Dnm-CAR) was constructed by inserting the truncated human TGFβ receptor I (TGFβRI, UniProt P36897) and TGFβ receptor II (TGFβRII, UniProt W8DXL6) sequences, lacking the intracellular kinase domains (as originally reported by Wieser et al. [34]) and separated by a T2A self-cleaving peptide, into the JK11 CAR by InFusion cloning. The JK59 and JK69 switch receptor (SwR)-STEAP1 CARs were constructed by inserting the extracellular domain of TGFβRI linked in frame with the transmembrane domain (TM) and intracellular domain (ICD) of the β chain of IL-2/IL-15 receptor (IL-2Rβ), a T2A self-cleaving peptide, and the extracellular domain of the TGFβRII linked in frame with the TM and ICD of the common γ chain of IL-2 receptor (IL-2Rγ) upstream of the P2A sequence by InFusion cloning. For the JK15 STEAP1-IgG CAR and JK69 SwR-IgG-STEAP1 CAR, the STEAP1-scFv sequence is linked to a human IgG1 hinge-CH2CH3 and transmembrane domain.

### 2.4. Retroviral Vector Production

Retroviral vectors were prepared by transient transfection of Phoenix-AMPHO cells using X-tremeGENE 9 DNA transfection reagent (Roche, Oslo, Norway) according to the manufacturer’s protocol. Briefly, 5.5 × 10^6^ phoenix-AMPHO cells were plated in 10 cm tissue-culture dishes. Then, 24 h later, cells were co-transfected with 3.7 µg of the retroviral plasmids, together with the packaging plasmids, and incubated at 37 °C overnight. Cells were then transferred to 32 °C, and the viral supernatants were harvested at 48 h and 72 h after transfection, cleared by centrifugation, and frozen down at −80 °C.

### 2.5. CAR T Cell Production and Expansion

T cells were activated with anti-CD3/CD28 antibodies and cultured with 100 IU/mL rhIL-2 for 2 days. Activated T cells were transduced with CAR retroviral supernatants on 24-well plates pre-coated with Retronectin (Takara Bio, Göteborg, Sweden, T100B-TAK). For JK11, JK15, CD19 CAR, and Dnm-CAR transductions, 0.3 × 10^6^ of activated T cells, resuspended in 1 mL of culture media supplemented with 200 IU/mL rhIL-2, were infected with 1 mL of retroviral supernatants, centrifuged at 900× *g* for 60 min at 32 °C and incubated at 37 °C. Then, 2 days later, 1 mL of media was replaced with fresh media supplemented with 100 IU/mL rhIL-2, and transduced T cells were expanded for 4 more days. For JK59 and JK69 CAR transductions, 500 µL of retroviral supernatant was added on Retronectin-coated plates, centrifuged at 2000 g for 60 min at 32 °C, removed, and then 0.3 × 10^6^ of activated T cells were added on the plates and infected as described for the other CARs. Then, 24 h later, the media was removed completely, and the same transduction method was performed 1 more time before media refreshment and expansion of CAR T cells. The expanded CAR T cells were frozen and stored in liquid N_2_. Before the functional assays, T cells were thawed and re-activated for 2 days with anti-CD3 and anti-CD28 antibodies and with 100 IU/mL rhIL-2. Alternatively, where noted, freshly transduced T cells were used directly for experiments.

### 2.6. TGFβ ELISA

Prostate cancer cell lines were cultured for 48 h in 10% FBS culture media. To reduce the background of TGFβ present in FBS, the cells were serum starved in media containing 0.5% of FBS for 24 more hours. Supernatants were harvested, centrifuged to remove any debris, and frozen down at −80 °C. The Human TGF-β1 Quantikine ELISA kit (R&D Systems, Abingdon, UK, DB100C) was used to quantify the amount of active TGFβ present in the supernatants and secreted by the prostate cancer cell lines, according to the manufacturer’s protocol.

### 2.7. Flow Cytometry Instruments, Staining, and Reagents

Flow cytometry analyses were performed on LSR II or Symphony instruments (BD Biosciences, Oslo, Norway), and data were analyzed using FlowJo™ Software (Tree Star Inc., Ashland, OR, USA). For all surface staining, cells were stained with the appropriate surface antibodies in the presence of the Fixable Viability Dye eFluor™ 780 (Thermo Fisher Scientific, Oslo, Norway, 65-0865) to label the dead cells. JK11, JK15, or CD19 CAR expression was determined using RQR8-binding mAbs QBend10-AlexaFluor^®^488 (FAB7227G) or QBend10-PE (FAB7227P) (R&D Systems, Abingdon, UK). Dnm, JK59, and JK69 CAR expression was determined using a TGFβRII-APC REAfinity™ antibody (Miltenyi Biotec, Lund, Sweden, 130-115-025). The CAR expression of the JK15 and JK69 CARs was determined using the R-Phycoerythin AffinityPure goat-anti-human IgG (H + L) secondary antibody (Jackson ImmunoResearch, Cambridge, UK, 109-116-088). For surface staining in the functionality assay, the following antibodies were used: CD3-Pacific Blue (317314), CD4-BV510 (741182), and CD8-BV711 (301044) (all from Biolegend, Oslo, Norway). To detect the intracellular cytokines, T cells were fixed and permeabilized in CytoFix/Cytoperm solution (BD Biosciences, Oslo, Norway, 554714), according to the manufacturer’s protocol, and stained with TNFα-PE (BD Biosciences, Oslo, Norway, 557068) and IFNγ-PECy7 (Thermo Fisher Scientific, Oslo, Norway, 25-73-19-82) antibodies. For the phenotyping of CAR T cells, the following antibodies were used: CD3-PerCPCy5.5 (317336), CD4-BV510 (741182), CD8-FITC (301050), CD25-BV605 (302632), LAG3-PECy7 (369310), TIM3-BV711 (345024), TIGIT-BV421 (372710), CCR7-PEDazzle (353236) (all from Biolegend, Oslo, Norway), CD45RA-BUV496 (741182), and PD1-BV786 (563789) (both from BD Biosciences, Oslo, Norway). To assess the cell surface expression of STEAP1, prostate cancer cell lines were stained with the anti-STEAP1 mouse antibody (mAb) generated in our laboratory [22], followed by the AlexaFluor^®^ 647 AffinityPure goat-anti-mouse IgG (H + L) secondary antibody (Jackson ImmunoResearch, Cambridge, UK, 115-605-003).

### 2.8. Functionality Assays (Killing and Cytokines Assay)

CAR T cells were, when specified, used freshly transduced, as described above, or thawed and re-activated with anti-CD3/CD28 antibodies for 2 days with 100 IU/mL rhIL-2. For the killing assays, CAR T cells pre-labeled with CellTrace™ Violet (Thermo Fisher Scientific, Oslo, Norway, C34557) were co-cultured with target cells, at the specified Effector-to-Target (E:T) ratios, for 24 h or 48 h. The GaspGLOW™ Fluorescein active Caspase-3 (FITC-DEVD-FMK) (Thermo Fisher Scientific, Oslo, Norway, 88-7004-42) or the cleaved caspase-3 (Red-DEVD-FMK) (Abcam, Cambridge, UK, ab65617) was added into the co-culture to measure the target cell caspase 3 activation by flow cytometry. Cell death was detected using the Fixable Viability Dye eFluor™ 780. Where indicated, recombinant human TGFβ (rhTGFβ) was added to the co-culture.

To measure TNFα and IFNγ production, CAR T cells were co-cultured with target cells at an E:T ratio of 1:3 for 18 h in the presence of BD GolgiPlug™ (555029) and BD GolgiStop™ (554724) protein transport inhibitors (BD Biosciences, Oslo, Norway), according to the manufacturer’s protocol. TNFα^+^ and IFNγ^+^ T cells were measured with flow cytometry using the specific antibodies.

### 2.9. Proliferation Assay in Response to TGFβ

T cells were activated and stimulated for 2 days with CD3/CD28 antibodies and transduced with the specific CAR constructs. On the day of the transduction (day 0), T cells were incubated with human recombinant TGFβ1 (rhTGFβ) (R&D System, Abingdon, UK, 7754-BH) at 1, 2.5, 5, 10, or 15 ng/mL. Media-containing rhTGFβ was refreshed twice a week until the end of the experiment. T cell proliferation was measured every 7 days for 3 weeks using 123 count eBeads™ (Thermo Fisher Scientific, Oslo, Norway, 01-1234-42) and analyzed with flow cytometry according to the manufacturer’s instructions. At day 21, CAR T cells were used in a 48 h killing assay at an E:T ratio of 1:1 with 22Rv1 target cells.

### 2.10. Long-Term Co-Culture Assays

CAR T cells were thawed and re-activated with anti-CD3/CD28 antibodies for 2 days with 100 IU/mL rhIL-2. Then, 22Rv1 and 22Rv1-KO target cells were irradiated at 20 Gy and plated at 0.6 × 10^6^ cells/well in 12-well plates. Then, 24 h later, 0.6 × 10^6^ CAR T cells (E:T ratio 1:1) were co-cultured with irradiated target cells in the presence or absence of 10 ng/mL rhTGFβ and without any additional rhIL-2 (day 0). Then, 3 days later (day 3), T cells from these cultures were collected, labeled with trypan blue, and counted using a Countess™ Automated Cell Counter (Thermo Fisher Scientific, Oslo, Norway). They were then re-plated on freshly-irradiated target cells at an E:T ratio of 1:1 in the presence or absence of 10 ng/mL rhTGFβ. This assay was repeated every 3–4 days until day 13 (i.e., day 6, day 10, and day 13). At days 0, 6, and 13, the expression of checkpoint markers on CAR T cells was measured with flow cytometry. At day 0 and day 13, CAR T cells were used in a 24 h killing assay at an E:T ratio of 1:1 with 22Rv1 target cells. Before each new co-culture, supernatants were collected and stored at −80 °C for multiplex cytokine analysis.

Irradiated C4-2B shGFP and C4-2B shSTEAP1, at 20 Gy, were co-cultured with freshly transduced CAR T at an E:T ratio of 1:1 in the presence or absence of 5 ng/mL rhTGFβ and without any additional rhIL-2 (day 0). Then, 7 days later, T cells from these cultures were collected, labeled with trypan blue, and counted as described for the 22Rv1 cultures. The T cells were then added to freshly irradiated C4-2B at an E:T ratio of 1:1 in the presence or absence of 5 ng/mL rhTGFβ. This assay was repeated every 7 days until day 28. Media-containing rhTGFβ was refreshed every 3 days with 5 ng/mL rhTGFβ. At day 18, supernatants were collected and stored at −80°C for further multiplex cytokine analysis. Each co-culture from each donor was performed in duplicate, and each duplicate pair was kept separate until the end of the experiment.

### 2.11. Multiplex Cytokine Analysis

Frozen supernatants from the long-term co-culture assay, collected at day 3, day 6, and day 13 (from 22Rv1 and 22 Rv1-KO target cells co-cultures), and at day 18 (from C4-2B shGFP and C4-2B shSTEAP1 target cells co-cultures), were thawed, and the secreted cytokines and chemokines were quantified using the Bio-Plex Pro Human Immunotherapy panel, 20-plex (Bio-Rad, Oslo, Norway, 12007975) according to the manufacturer’s instructions. Samples were measured on a Luminex 200 instrument system (Bio-Rad, Oslo, Norway) and evaluated using the Bio-Plex Manager^™^ software, version 6.2. Supernatants from each donor were analyzed in duplicate. Each duplicate pair was kept separate from T cell stimulation until the end of the experiment.

### 2.12. Statistical Analysis

All statistical analysis was performed using GraphPad Prism v9 (GraphPad Software Inc.). The differences among groups for all experiments were determined with a one-or two-way ANOVA followed by Tukey’s multiple comparisons test. *p*-values < 0.05 were considered significant.

## 3. Results

### 3.1. Design of TGFβ-SWITCH Receptor and Co-Expression with STEAP1 CAR 

**Figure 1:**
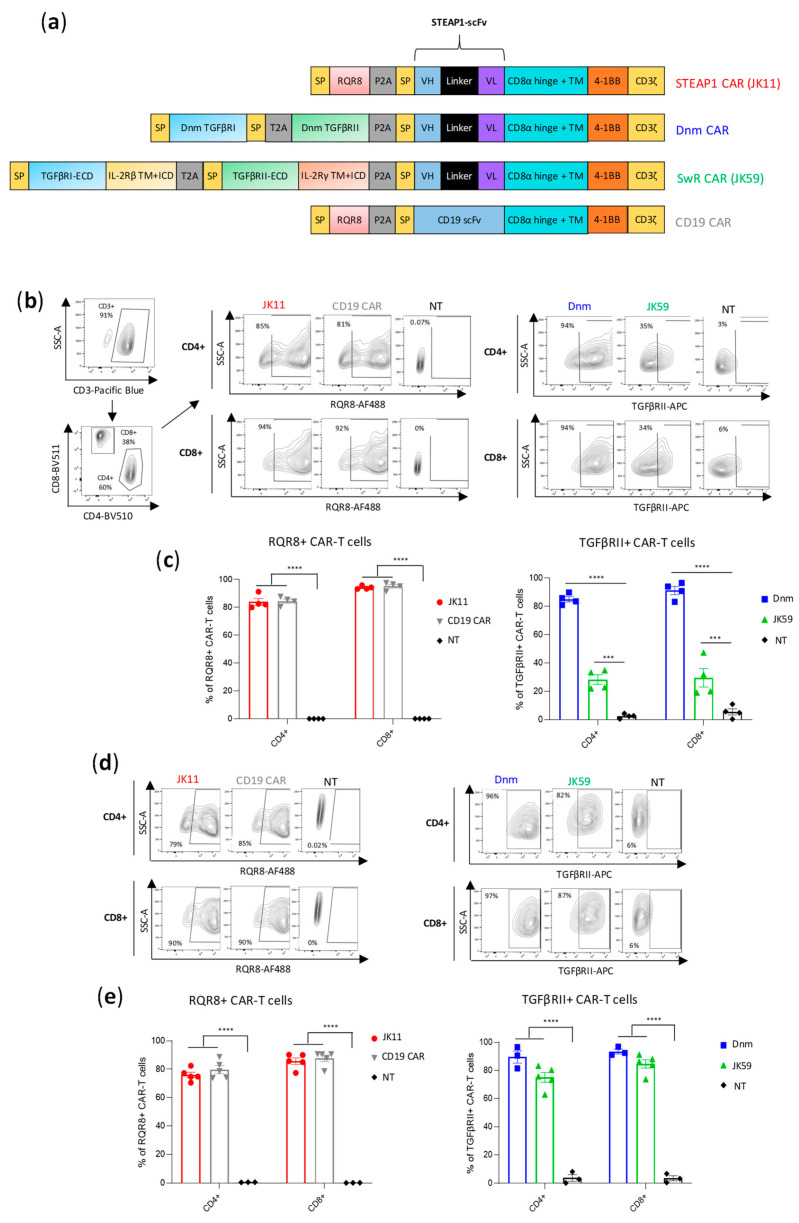
CAR design and expression. (**a**) Schematic representations of the CAR constructs; parental STEAP1 CAR (JK11), control CD19 CAR, dominant negative mutant -STEAP1 CAR (Dnm) with the TGFβ receptor (TGFβR) lacking the intracellular signaling domain, TGFβ-switch receptor (SwR)-STEAP1 CAR (JK59) with the extracellular domain (ECD) of the TGFβRI and TGFβRII subunits fused to the transmembrane domain (TM) and intracellular domain (ICD) of the IL-2/IL-15 receptor β and γ chains, respectively (IL-2Rβ and IL-2Rγ). (**b**) Flow analysis of CAR expression in primary human T cells stained with CD3, CD4, and CD8 (left panel), and with the antibody recognizing the RQR8 sequence of JK11 and CD19 control CAR (middle panel) or the TGFβRII antibody recognizing Dnm and JK59 CARs (right panel). T cells were stained five days after viral transduction. (**c**) Graphs representing the expression of JK11 and CD19 CARs (right panel) and Dnm and JK59 CARs (left panel). Data represent the mean values ± SEM of 4 different T cell transductions from 4 different healthy donors (each transduction was performed in duplicate). (**d**) Flow analysis of the CAR expression in human T cells after CAR T cells freezing/thawing and two days activation with anti-CD3/CD28 antibodies. Contour plots of JK11 and CD19 CAR (left panel) and of Dnm and JK59 CAR expression (right panel). (**e**) Graphs representing the expression of JK11 and CD19 CARs (left panel) and Dnm and JK59 CARs (right panel) after freeze-thawing and activation for two days, from three to five healthy donors. Data represent the mean values ± SEM. Data were analyzed by one-way ANOVA with Tukey’s multiple comparisons test. *** *p* < 0.001; **** *p* < 0.0001.

We analyzed the structure of the TGFβ receptor (TGFβR), which consists of multiple subunits, and compared it to the intracellular domains (ICD) of different stimulatory receptors. Based on this, we selected the ICD of the IL-2/IL-15 receptor β and γ chains as a good candidate for a switch receptor (SwR), intended to be triggered by TGFβ but conferring a stimulatory signal to the T cells. We designed a TGFβ-SwR, encompassing the extracellular domain (ECD) of the TGFβRI and TGFβRII subunits fused to the transmembrane domain (TM) and ICD of the IL-2/IL-15 receptor β and γ chains, respectively (Figure 1a). The TGFβ-SwR was cloned into our anti-STEAP1 CAR construct JK11, which incorporates the co-stimulatory 41BB domain. The resulting SwR-CAR construct was called JK59. For use as reference, we made a dominant negative mutant (Dnm) lacking the intracellular domain of the TGFβR and cloned this into the JK11 STEAP1 CAR. CAR constructs may confer a level of target-independent activity. As a control, we, therefore, employed a CAR comprising a CD19-specific scFv, cloned into the same backbone as the JK11 STEAP1 CAR, with identical spacer, transmembrane, and intracellular domains (CD8 spacer/TM domain, 4-1BB, CD3ζ) (Figure 1a).

The expression of the JK11 STEAP1 CAR (RQR8-STEAP1_CD8_4-1BBζ) and CD19 CAR after PBMC transduction was measured with flow cytometry, using a mAb against the marker gene RQR8 [35]. The expression of JK59 (SwR-STEAP1_CD8_4-1BBζ) and the Dnm-CAR (Dnm-STEAP1_CD8_4-1BBζ) was measured with a mAb against TGFβRII (Figure 1b,c). Across four different donors, the mean JK59 expression was 28% in CD4^+^ T cells and 30% in CD8^+^ T cells. The Dnm-CAR was expressed in > 80% of primary T cells, as were the JK11 and the CD19 CARs. The phenotype of the CAR T cells was characterized by flow cytometry nine days post-transduction. Figure S1a shows the expression of activation/exhaustion markers from three healthy donors in JK11, Dnm, JK59, and CD19 CAR T cells. No differences were observed between the CAR T cells groups, with 50–54% CD25^+^ CD4^+^ T cells, 25–28% of PD1^+^CD4^+^ T cells, 15–27% of TIGIT^+^CD4^+^ cells, and almost no LAG3 or TIM3 positive CD4^+^ T cells. The CD8^+^ T cells were 54–71% CD25^+^, 9–18% PD1^+^, 13–37% LAG3^+^, 7–20% TIM3^+,^ and about 32% TIGIT^+^.

The T cell yield after transduction and expansion was similar between JK11, Dnm, and CD19 CAR T cells, with a 5 to 6-fold increase in the number of live cells. However, the number of live JK59-transduced T cells was higher than JK11, Dnm, and CD19 T cells, with a 14-fold expansion.

T cells for clinical use need to be frozen, thawed, and re-stimulated before infusion into patients. Interestingly, the expression of JK59 was markedly increased after freezing/thawing and two days of re-stimulation, with a mean of 75% and 74% in CD4^+^ and CD8^+^ T cells, respectively (Figure 1d,e). The expression of JK11, the Dnm CAR, and the CD19 CAR was largely unchanged, at 70–90%. The viability of CAR T cells before cryopreservation and after thawing and 2 days of re-activation was mostly unchanged in JK11, Dnm, and CD19 CAR T cells, while a small decrease was observed in the JK59 SwR CAR T cells (Figure S1b). In addition, we observed an important decrease in the cell recovery in the JK59 CAR T cells during the cryopreservation process, with a 35% recovery yield, when compared to JK11 (66%), Dnm (80%), and CD19 (67%) CAR T cells (Figure S1c).

The expression level described above may not be directly comparable between the constructs, as the expression was detected with different mAbs, binding to different proteins (RQR8, TGFβR). To obtain a comparable and direct CAR detection and to assess if modifications to the spacer may affect the functionality, we cloned CAR constructs with an alternative spacer and TM domain derived from IgG1 but with mutations to avoid FcR binding [36] (Figure S2a). In these constructs, the expression of the CAR protein could be directly measured with a mAb against the IgG-spacer and compared between the constructs. The IgG-stainings demonstrated that the RQR8-STEAP1_IgG CAR construct (JK15) was expressed in 80% of transduced T cells, whereas the SwR-STEAP1_IgG CAR (JK69) was expressed in 40% of T cells (Figure S2b,c). The recorded percentages from IgG-stainings were in line with measurements based on mAbs against RQR8 and TGFβR (Figure S2b,c). Even for these constructs with IgG-spacers, the expression of the SwR-CAR (JK69) increased substantially after freezing/thawing, while the expression of the stand-alone CAR (JK15) was largely unchanged (Figure S2d,e).

### 3.2. STEAP1 Expression and TGFβ Secretion in Target Cell Lines

The functionality of the JK59 and Dnm-CAR constructs was investigated by using STEAP1^+/−^ prostate cancer cell lines as targets. We have previously determined the STEAP1 expression in these cell lines [22]. As the expression levels may change during culture, new flow cytometry stainings of the cultured cells were performed. Cell lines 22Rv1 and LNCap were highly STEAP1^+^, whereas cell lines PC3 and DU145 were confirmed to be STEAP1 negative, as was a CRISPR/Cas STEAP1 knockout variant of 22Rv1 (22Rv1-KO) (Figure S3a). Further, we generated 22Rv1 cells stably overexpressing TGFβ (22Rv1-TGFβ), which was co-expressed with EGFP. The TGFβ-EGFP construct was expressed in > 98% of the 22Rv1-TGFβ cells (Figure S3b), and STEAP1 expression was retained (Figure S3a). Figure S3c shows the level of TGFβ secretion in 22Rv1, LNCaP, C4-2B, PC3, DU145, and 22Rv1-TGFβ cultures. 

### 3.3. SwR STEAP1 CAR T Cells Exhibit Target-Specific Cytokine Response

**Figure 2:**
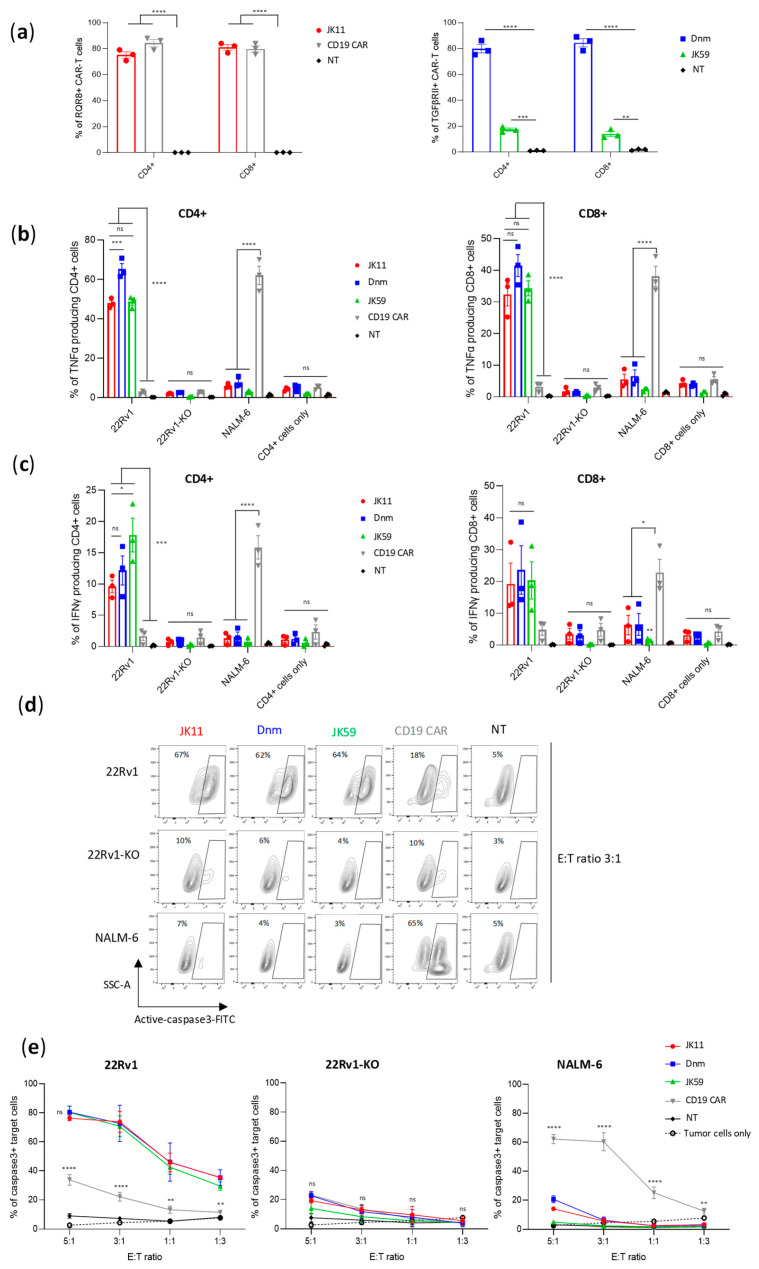
Dnm and SwR-STEAP1 CAR T cells induce the production of IFNγ and TNFα and apoptosis upon co-culture with STEAP1^+^ target cells in vitro. (**a**) Graphs of the CAR expression (measured five days after transduction) of freshly transduced JK11, Dnm, JK59, and CD19 CAR T cells and non-transduced (NT) T cells. The CAR expression of JK11 and CD19 CAR T cells (left panel) was detected with the antibody recognizing the RQR8 sequence, and the CAR expression of Dnm and JK59 CAR T cells (right panel) was detected with the TGFβRII antibody (as shown in Figure 1). Data represent mean values ± SEM of three different healthy donors, each in triplicate. Data were analyzed by two-way ANOVA with Tukey’s multiple comparisons test. ** *p* < 0.01; *** *p* < 0.001; **** *p* < 0.0001. (**b**,**c**) CAR T cells and non-transduced (NT) cells were co-cultured (in duplicate) with 22Rv1, 22Rv1-KO, and NALM-6 target cells at an E:T ratio of 1:3 for 18 h. Production of TNFα and IFNγ was measured with flow cytometry by gating on CD3^+^, and CD4^+,^ or CD8^+^ cells (gating strategy in Figure S4a–c). (**b**) Percentage of TNFα production in CD4^+^ (left panel) and CD8^+^ (right panel) T cells. (**c**) Percentage of IFNγ production in CD4^+^ (left panel) and CD8^+^ (right panel) T cells. CAR T cells cultured alone (CD4^+^ and CD8^+^ cells only) were included as controls to indicate the baseline of TNFα (in (**b**)) and of IFNγ (in (**c**)). Data represent mean values ± SEM of three different healthy donors, each in duplicate. Graphs are representative of two independent experiments. Data were analyzed by one-way ANOVA with Tukey’s multiple comparisons test. * *p* < 0.05; *** *p* < 0.001; **** *p* < 0.0001; ns = not significant. (**d**) Representative contour plots of 22Rv1, 22Rv1-KO, and NALM-6 target cells co-cultured with freshly transduced CAR T cells or non-transduced (NT) cells, at an E:T ratio of 3:1 for 24 h. Lysis of target cells was measured by the intensity of FITC-active-caspase3 using flow cytometry (gating strategy in Figure S4d). (**e**) Graphs representing the percentage of apoptotic cells among 22Rv1 (left panel), 22Rv1-KO (middle panel), and NALM-6 (right panel) cells co-cultured with the different CAR T cell groups, or the NT control group at E:T ratios 1:3, 1:1, 3:1, and 5:1 for 24 h. Target cells cultured alone (tumor cells only) were included as controls to indicate the baseline of active caspase3. Data represent mean values ± SEM of two different healthy donors, each in duplicate. Graphs are representative of two independent experiments. Data were analyzed by two-way ANOVA with Tukey’s multiple comparisons test. ** *p* < 0.01; **** *p* < 0.0001; ns = not significant.

Figure 2 shows a representative flow cytometry experiment assessing the TNFα and IFNγ response. Primary T cells were transduced with different CAR constructs (JK11, Dnm, JK59, CD19 CAR; all with CD8 spacer) or left non-transduced (NT) and then co-cultured overnight with STEAP1 positive or negative target cells. The proportion of responding T cells was determined as shown in Figure S4b (TNFα) and Figure S4c (IFNγ), based on the gating strategy shown in Figure S4a. We found that all three STEAP1 CAR T cell populations (JK59, Dnm, JK11) responded with the production of TNFα and IFNγ, and that this response was dependent both on the expression of STEAP1 in target cells and on the STEAP1-specific scFv (compared to CD19-specific scFv). For all three STEAP1 CAR T variants, the percentage of TNFα^+^ T cells was higher for CD4^+^ (~50–70%) than CD8^+^ cells (~20–50%) (Figure 2b), while the IFNγ^+^ percentage was higher for CD8^+^ cells (Figure 2c). The functionality of the CD19 CAR control was confirmed, as the CD19 CAR T cells responded to CD19^+^ NALM-6 leukemia targets. Intriguingly, the percentage of TNFα^+^ and IFNγ^+^ T cells was similar for JK59 and the reference JK11 (Figure 2b,c), even though the percentage of T cells expressing the SwR-CAR construct was only about 20% compared to the parental JK11 CAR in these freshly transduced cells (Figure 2a). This observation was made in repeated independent experiments with T cells from three donors. In most experiments, the percentage of TNFα^+^ T cells was highest for the Dnm-CAR, while the IFNγ response was similar for the JK59, Dnm, and JK11 CAR.

### 3.4. SwR STEAP1 CAR T Cells Specifically Kill Target Cells

Next, we investigated the ability of the CAR T cells to kill tumor cells by assessing the induction of apoptosis in target cells, as measured by active caspase-3 (aCasp3). The different STEAP1 CAR T cells (JK59, Dnm, and JK11), as well as control T cells (CD19 CAR, NT) were co-cultured with STEAP1^+/−^ tumor cells at different effector-to-target (E:T) ratios. Figure S3a shows STEAP1 expression in target cells. The proportion of apoptotic (aCasp3^+^) target cells was determined as shown in Figure 2d, based on the gating strategy shown in Figure S4d. We found that the induction of apoptosis was significantly increased in STEAP1^+^ targets co-cultured with the JK59 and Dnm CAR T cells, compared to the CD19 CAR and NT controls (Figure 2d,e). The proportion of aCasp3^+^ targets was similar to the level obtained with JK11 STEAP1 CAR, indicating a fully retained killing capacity, even though the percentage of T cells expressing the CAR was lower (Figure 2a). The killing increased, as expected, with increasing E:T ratios and reached 80% at an E:T ratio of 5:1 (Figure 2e). The killing was STEAP1-specific, as no significant differences between the STEAP1 and CD19 CAR T cells were observed for 22Rv1-KO targets, and only CD19 CAR T cells killed NALM-6 target cells (Figure 2d,e).

### 3.5. SwR Improves the Cytotoxic Capacity of STEAP1 CAR T Cells in a TGFβ-Rich Environment 

**Figure 3:**
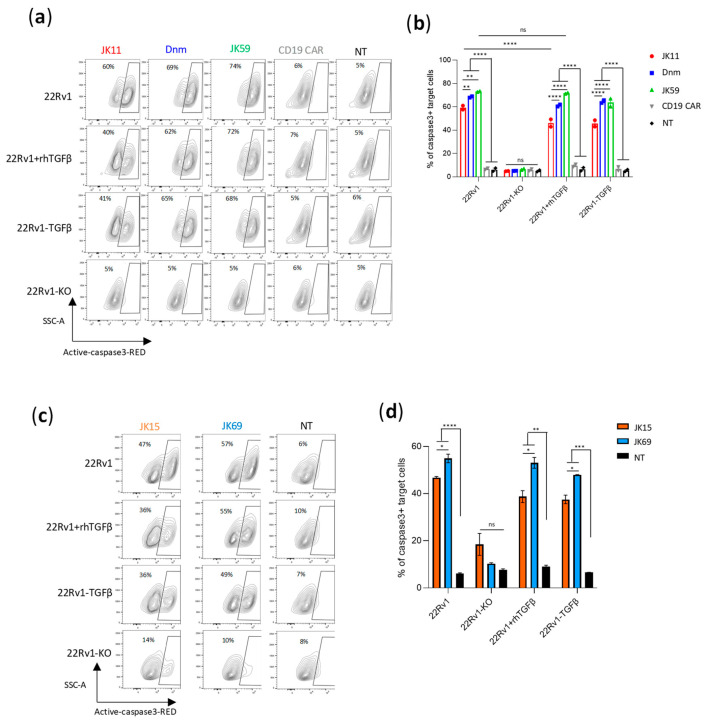
SwR-STEAP1 CAR T cells have superior cytotoxic activity in a TGFβ-rich environment. (**a**) Representative contour plots of 22Rv1, 22Rv1-KO, and 22Rv1-TGFβ target cell lines, and 22Rv1 target cells in the presence of 10 ng/mL rhTGFβ, co-cultured with freeze-thawed and non-activated JK11, Dnm, JK59, and CD19 CAR T cells, or NT T cells, at an E:T ratio of 1:1 for 48 h. Lysis of target cells was measured by the intensity of Red-active-caspase3, using flow cytometry. (**b**) A graph representing the percentage of apoptotic cells among 22Rv1, 22Rv1-KO, and 22Rv1-TGFβ cell lines and 22Rv1 cells cultured in the presence of 10 ng/mL rhTGFβ. Data represent the mean values ± SEM of two healthy donors from one representative experiment, in which each co-culture was performed in duplicate. The experiment was repeated two times. Data were analyzed by two-way ANOVA with Tukey’s multiple comparisons test. ** *p* < 0.01; **** *p* < 0.0001; ns = not significant. (**c**) Representative contour plots of 22Rv1, 22Rv1-KO, 22Rv1-TGFβ target cell lines, and 22Rv1 target cells in the presence of 10 ng/mL rhTGFβ, co-cultured with frozen/thawed and non-activated JK15 and JK69 CAR T cells, or non-transduced (NT) T cells, at an E:T ratio of 1:1 for 48 h. Lysis of target cells was measured by the intensity of Red-active-caspase3 staining/labeling by flow cytometry (gating strategy in Figure S4d). (**d**) A graph representing the percentage of apoptotic cells among 22Rv1, 22Rv1-KO, 22Rv1-TGFβ cell lines, and 22Rv1 cells cultured in the presence of 10 ng/mL rhTGFβ. Data represent the mean values ± SEM of one healthy donor (in duplicates) from one representative experiment. The experiment was repeated two times. Data were analyzed by one-way ANOVA with Tukey’s multiple comparisons test. * *p* < 0.05; ** *p* < 0.01; *** *p* < 0.001; **** *p* < 0.0001; ns = not significant.

We hypothesized that the JK59 SwR-CAR T cells would show superior functionality in a TGFβ-rich environment. To investigate this hypothesis, different CAR T cell variants were co-cultured with STEAP1^+/−^ target cells, supplemented or not with rhTGFβ, and the induction of apoptosis was analyzed with flow cytometry (Figure 3a,b). The experiment demonstrated that all three STEAP1 CAR T cells killed target cells in a STEAP1-dependent manner, even in the presence of TGFβ. The JK59 CAR and Dnm-CAR gave significantly higher levels of killing compared to the parent JK11 CAR. This difference was more prominent for the 22Rv1 cell line overexpressing TGFβ (22Rv1-TGFβ), as compared to wild-type 22Rv1 cells, and for the cultures supplemented with rhTGFβ. For the JK11 CAR, the observed level of killing was lower in the cultures that contained TGFβ. The results were, thus, consistent with the hypothesis that the JK59 CAR T cells have superior functionality in a TGFβ-rich environment. A similar pattern was observed for the STEAP1 CAR with an IgG-based spacer. The JK69 CAR conferred T cells with a higher cytotoxic activity than the parent JK15 CAR, and the difference appeared most prominent for TGFβ-rich cultures (Figure 3c,d).

### 3.6. SwR Gives Improved Expansion of STEAP1 CAR T Cells in a TGFβ-Rich Environment 

**Figure 4:**
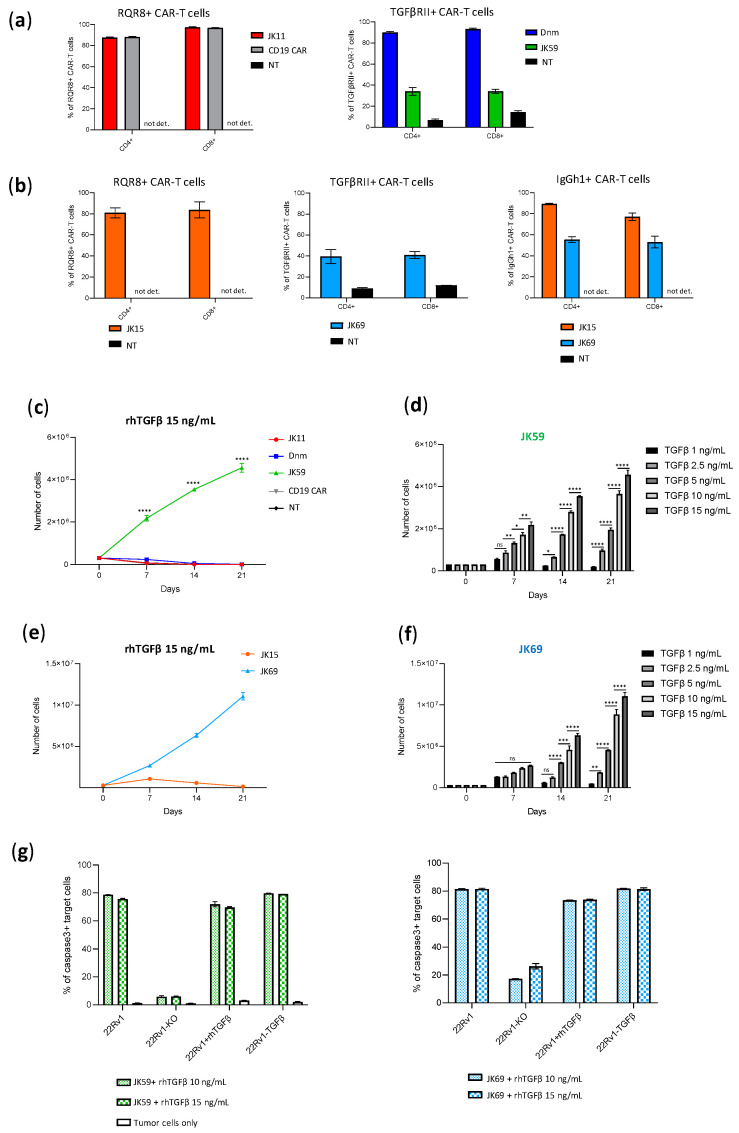
TGFβ induces proliferation of the SwR-STEAP1 CAR T cells. (**a**) Graphs representing the CAR expression (measured six days after transduction) of freshly transduced JK11, Dnm, JK59, and CD19 CAR T cells and non-transduced (NT) T cells. The CAR expression of JK11 and CD19 CAR T cells was detected with the antibody recognizing the RQR8 sequence (left panel), and the CAR expression of Dnm and JK59 CAR T cells was detected with the TGFβRII antibody (right panel) (as shown in Figure 1). not det.: not detected (**b**) Graphs representing the CAR expression (measured six days after transduction) of freshly transduced JK15 and JK69 CAR T cells. The CAR expression was detected with the antibody recognizing the IgGh1 hinge of JK15 and JK69 CARs (right panel) (as shown in Figure S2). The CAR expression was also detected with the antibody recognizing the RQR8 sequence (left panel) and with the TGFβRII antibody (middle panel) in the JK15 and JK69 CAR T cells, respectively. not det: not detected. (**c**,**e**) The freshly transduced CAR T cells, and non-transduced (NT) T cells, were cultured for 21 days in the presence of 15 ng/mL recombinant human TGFβ (rhTGFβ). The absolute number of T cells was measured every 7 days (from day of transduction, day 0) with flow cytometry using counting beads. In (**c**), CD19 and NT lines follow the JK11 line and are, therefore, not visible. (**d**,**f**) The number of JK59 and JK69 CAR T cells measured every 7 days with flow cytometry using counting beads, after incubation with different concentrations of rhTGFβ, until day 21. Data represent the mean values ± SEM of one healthy donor (in duplicates) from one representative experiment. The experiment was repeated two times. Data were analyzed by one-way ANOVA with Tukey’s multiple comparisons test. * *p* < 0.05; ** *p* < 0.01; *** *p* < 0.001; **** *p* < 0.0001; ns = not significant. (**g**) At the end of the proliferation assay (day 21), JK59 (left panel) and JK69 (right panel) CAR T cells cultured with 10 ng/mL and 15 ng/mL rhTGFβ were co-cultured with 22Rv1, 22Rv1-KO, 22Rv1-TGFβ cell lines and 22Rv1 cells cultured in the presence of 10 ng/mL rhTGFβ (22Rv1 + rhTGFβ) at an E:T ratio 1:1 for 48 h. Lysis of target cells was measured by the intensity of Red-active-caspase3, using flow cytometry (gating strategy in Figure S4d). The percentage of apoptotic target cells is represented in the graphs. Data represent the mean values ± SEM of one healthy donor (in duplicates) from one experiment.

We further investigated how the two SwR-CAR T cell variants (JK59 and JK69) responded to TGFβ, compared to the Dnm-CAR, the JK11 reference, and the controls. To this aim, the proliferative capacity and survival of the T cells were monitored for 21 days, where the T cells were cultured with TGFβ at concentrations ranging from 1.0–15 ng/mL. As shown in Figure 4, the number of SwR-STEAP1 CAR T cells increased steadily over the entire observation period, resulting in a 15-fold expansion for JK59 and a 33-fold expansion for JK69, whereas none of the other CAR T cell groups showed any expansion at any time point (Figure 4c,e). After 21 days, the SwR-STEAP1 CAR T cells still expanded in numbers, while no viable cells were detected for any of the other T cell populations. This applied to both SwR variants (JK59, Figure 4c,d; JK69, Figure 4e,f). The effect of TGFβ on the SwR CAR T cells was dose-dependent and increased gradually over the range of 1.0–15 ng/mL TGFβ (Figure 4d,f).

Next, we asked if the functionality of the SwR-STEAP1 CAR T cells was retained after long-term expansion in a TGFβ-rich environment and if their functionality was impacted by the continued presence of TGFβ during their encounter with target cells. The two SwR-STEAP1 CAR T cell variants were harvested after 21 days and co-cultured with STEAP1^+/−^ tumor cells (wild-type 22Rv1 and 22Rv1-KO), as well as with 22Rv1 cells overexpressing TGFβ (22Rv1-TGFβ) or supplemented with rhTGFβ (22Rv1+rhTGFβ). As shown in Figure 4g, the assay indicated that both JK59 and JK69 had retained STEAP1-specific cytotoxic activity, giving induction of apoptosis in a high proportion (80%) of target cells. The continued presence of TGFβ did not affect the level of killing.

### 3.7. CAR T Cell Expansion and Functionality upon Repeated Target Cell Exposure 

**Figure 5:**
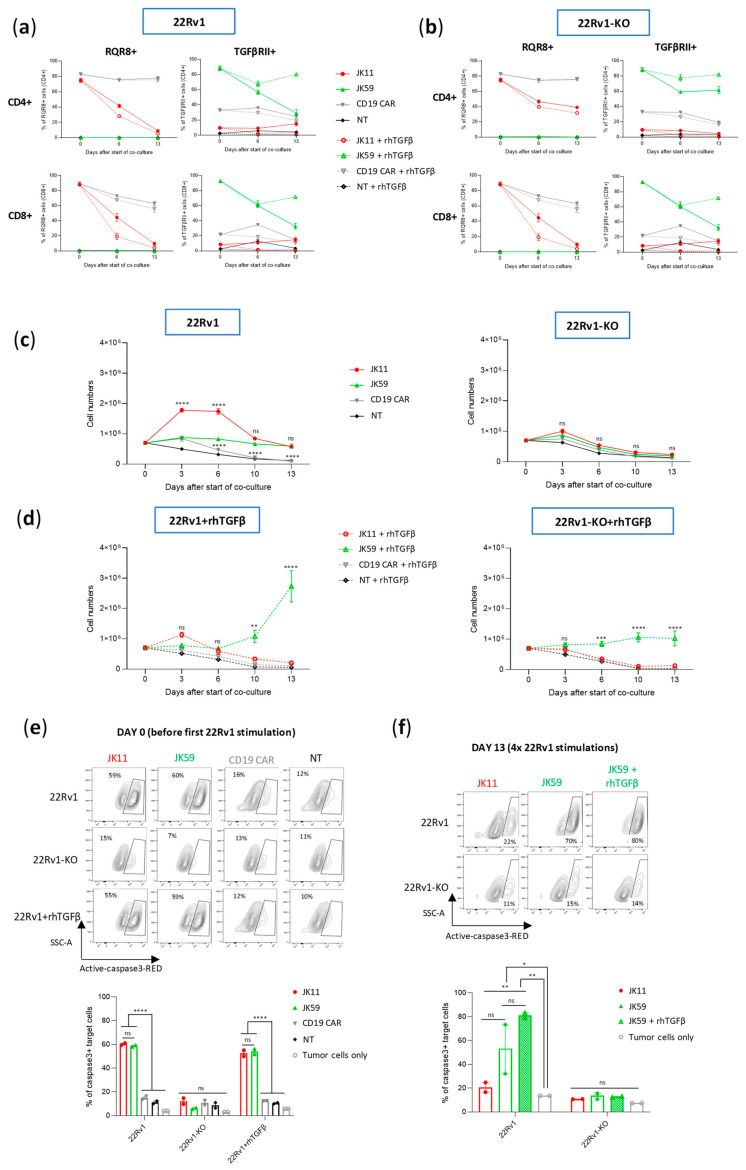
TGFβ enhances the proliferation and functionality of SwR-STEAP1 CAR T cells after repeated in vitro stimulation with tumor cells. Freeze-thawed and activated JK11, JK59, and CD19 CAR T cells, and non-transduced (NT) control T cells, were co-cultured with irradiated (20 Gy) 22Rv1 and 22Rv1-KO target cells in the absence or presence of 10 ng/mL rhTGFβ at an E:T ratio of 1:1 for 3 to 4 days. Every 3 to 4 days, the T cell number was measured, and the same T cells were co-cultured again on freshly irradiated target cells for 3 to 4 more days until day 13. (**a**) Graphs representing the CAR expression of JK11, JK59, and CD19 CAR T cells and non-transduced (NT) T cells following co-culture with 22Rv1 target cells at days 0, 6, and 13. (**b**) Graphs representing the CAR expression of JK11, JK59, and CD19 CAR T cells and non-transduced (NT) T cells following co-culture with 22Rv1-KO target cells at days 0, 6, and 13. In (**a**,**b**), the CAR expression of JK11 and CD19 CAR T cells was detected with the antibody recognizing the RQR8 sequence (left panels), and the CAR expression of JK59 CAR T cells was detected with the TGFβRII antibody (right panels) (**c**) A graph representing the number of T cells following co-culture with 22Rv1 (left panel) or 22Rv1-KO (right panel) target cells at days 0, 3, 6, 10, and 13. (**d**) A graph representing the number of T cells after co-culture with 22Rv1 (left panel) or 22Rv1-KO (right panel) target cells at days 0, 3, 6, 10, and 13 in the presence of 10 ng/mL rhTGFβ. Data in (**c**,**d**) represent the mean values ± SEM of two healthy donors. Each co-culture with each donor’s cells was performed in duplicate, and each duplicate pair was kept separate until the end of the experiment. Data were determined by two-way ANOVA with Tukey’s multiple comparisons test. ** *p* < 0.01; *** *p* < 0.001; **** *p* < 0.0001; ns = not significant. (**e**) Before the start of the long-term co-culture (day 0), JK11, JK59, and CD19 CAR T cells and NT T cells were co-cultured with 22Rv1 and 22Rv1-KO target cells, and with 22Rv1 in the presence of 10 ng/mL rhTGFβ at an E:T ratio of 1:1 for 24 h. (**f**) At the end of the long-term co-culture (day 13), JK11, JK59 CAR T cells, and JK59 CAR T cells cultured with 10 ng/mL rhTGFβ, each co-cultured with 22Rv1 target cells for 13 days, were co-cultured with 22Rv1 and 22Rv1-KO cells at an E:T ratio 1:1 for 24 h. In (**e**,**f**), lysis of target cells was measured by the intensity of Red-active-caspase3, using flow cytometry, as represented by the contour plots (top panels) (gating strategy in Figure S4d). The percentage of apoptotic target cells is represented in the graphs (lower panels). Target cells cultured alone (tumor cells only) were included as controls to indicate the baseline of active caspase3. Data represent the mean values ± SEM of two healthy donors. Each co-culture from each donor was performed in duplicate, and each duplicate pair was kept separate until the end of the experiment. In (**e**,**f**), statistical significances were determined by one-way ANOVA with Tukey’s multiple comparisons test. * *p* < 0.05; ** *p* < 0.01; **** *p* < 0.0001; ns = not significant.

It is known that continued antigen stimulation of CAR T cells may lead to T cell exhaustion or activation-induced cell death (AICD) [37]. The SwR-ICD was derived from a domain shared by the receptors for IL-2 and IL-15. While IL-15 is mainly considered to promote T cell survival, the continued stimulation of IL-2R may also give T cell exhaustion and AICD. We investigated how the CAR T variants responded to continuous/repeated antigen exposure by subjecting the T cells to 4 serial stimulations over 2 weeks with irradiated 22Rv1 cells or 22Rv1-KO controls. Throughout the assay, the T cells were kept in culture with the irradiated feeder cells, which were replaced every 3–4 days. At “day 0”, the cells had been thawed and re-activated with anti-CD3/CD28 antibodies. The experiment was performed with two donors, each in duplicates, with or without the addition of rhTGFβ. At day 0, the expression levels of the CAR constructs with/without SwR were similar (~80–90%), as estimated by staining for RQR8 or TGFβRII (Figure 5a,b). Cell counts from cultures without supplemented rhTGFβ showed that the JK11 and JK59 CAR T cells survived until the end of the experiment, while the NT and CD19 CAR T controls did not (Figure 5c). The number of JK11 cells increased during the first week but then subsided, while the JK59 CAR T cells maintained stable numbers. The survival and expansion were STEAP1-dependent, as no T cells survived in the 22Rv1-KO cultures. 

In cultures supplemented with rhTGFβ, the JK11 CAR T cells started decreasing in numbers after the second stimulation and did not survive until day 13. In contrast, the JK59 CAR T cells increased substantially in numbers after the third and fourth stimulation (Figure 5d). In the 22Rv1-KO cultures supplemented with rhTGFβ, the number of JK59 CAR T cells remained largely stable. The CAR T cell expansion was also evaluated in another assay, where freshly transduced JK11, Dnm, JK59, and CD19 CAR T cells were co-cultured with irradiated STEAP1^+^ C4-2B shGFP cells or C4-2B shSTEAP1 control cells for 28 days, with or without the addition of rhTGFβ. T cell counts were measured every 7 days after the addition of fresh irradiated target cells. As observed with the 22Rv1 target cells, the number of JK59 CAR T cells increased substantially (~75-fold) in C4-2B shGFP cultures supplemented with rhTGFβ, while the number of JK11 and Dnm CAR T cells slightly decreased (Figure S5b, left panel). In the C4-2B shSTEAP1 cultures with rhTGFb, JK59 CAR T cell number increased slightly (~2-fold) until day 28 (Figure S5b right panel), whereas the numbers of JK11 and Dnm CAR T cell decreased. The data, thus, indicated that the expansion of JK59 CAR T cells was dependent both on STEAP1 and TGFβ. CAR expression decreased considerably for JK11 over the 14 days (30–40% at day 6, 5–10% at day 13), while it remained relatively high for JK59 (~60% at day 6, 70–80% at day 13; Figure 5a,b). 

CAR T functionality was assessed with aCasp3 assays before the start of co-culture (day 0) and after the four serial stimulations (day 13). At day 0, the JK11 and JK59 CAR T cells both showed potent STEAP1-specific cytotoxic activity at similar levels (Figure 5e). At day 13, JK11 T cells cultured with rhTGFβ had not survived, while JK11 T cells cultured without rhTGFβ showed modest cytotoxic activity (Figure 5f). The SwR CAR T cells (JK59) showed substantially higher cytotoxic activity, and those cultured with rhTGFβ killed the largest proportion of target cells (~80%; Figure 5f).

**Figure 6:**
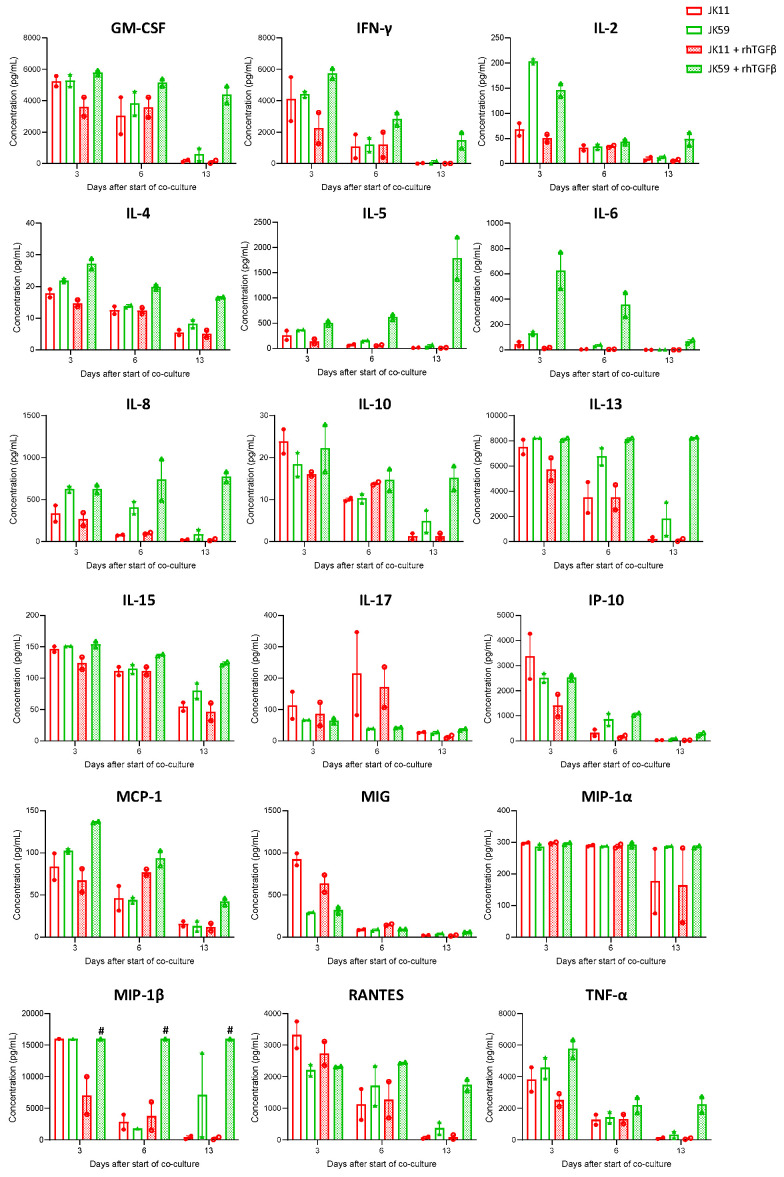
TGFβ maintains a high cytokine profile in SwR-STEAP1 CAR T cells after repeated in vitro stimulation of STEAP1^+^ tumor cells. Luminex cytokine analyses were performed using T cells supernatants isolated at days 3, 6, and 13 from JK11 and JK59 CAR T cells and non-transduced (NT) T cells co-cultured with 22Rv1 target cells in the absence or presence of 10 ng/mL rhTGFβ (as shown in Figure 5). Data represent the mean values ± SEM from two healthy donors. Each co-culture from each donor was performed in duplicate, and each duplicate pair was kept separate throughout the experiment. The “#” symbol indicates that the values are higher than 16,000 pg/mL.

The cytokine profile was characterized in multiplex assays (Figure 6). The initial evaluation at day 3 showed that the JK11 CAR T cells secreted a wide range of cytokines upon 22Rv1 stimulation and that this response was STEAP1-dependent (Figure S6). The cytokine response was Th1-weighted, with high levels of TNFα and IFNγ and only marginal levels of IL-4 and IL-10. The CAR T cells also secreted GM-CSF, IL-15, and the chemokines IL-8, IP-10, MIG, MIP-1α, MIP-1β, and RANTES, suggesting polyfunctionality and potential to attract and support immune cells. Interestingly, the supplementation of TGFβ gave an attenuated cytokine response for JK11 CAR T cells, while the JK59 CAR T cells were resistant to this suppression.

After repeated stimulations with target cells, the cytokine response subsided in the JK11 CAR T cells but persisted for the JK59 CAR T cells in the rhTGFβ-rich cultures (Figure 6). At day 13, the measured levels for most cytokines were minimal in JK11 CAR T cultures, even after 22Rv1 stimulation without rhTGFβ, where the number of viable T cells was comparable to day 0 (Figure 5c). In the JK59 CAR T cell cultures without supplemented rhTGFβ, most cytokines were still detected at day 13, whereas the levels were substantially higher in the TGFβ-rich cultures (Figure 6). Furthermore, cytokine profiling upon C4-2B shGFP stimulation at day 18 (three stimulations) showed that the Dnm-CAR T cells secreted more cytokines than JK11 and JK59 CAR T cells in cultures without rhTGFβ (Figure S5c). In TGFβ-rich culture, the secretion of cytokines increased significantly in JK59 cultures, while it remained stable in JK11 and Dnm cultures (Figure S5c).

Taken together, the results indicated that the effect of TGFβ was opposite on STEAP1 CAR T cells with/without expression of the SwR. The JK11 CAR T cells were suppressed by TGFβ, whereas the SwR CAR T cells (JK59) were stimulated, both regarding proliferation/survival, cytotoxicity, and cytokine secretion. The data after 14 days (four stimulations) suggested that the functionality of the JK59 CAR T cells was intact, with no evidence of exhaustion or AICD.

### 3.8. CAR T Cell Phenotype upon Repeated Target Cell Exposure 

We furthermore investigated, using flow cytometry, how the T cell phenotype evolved in the cultures described above upon stimulation four times with irradiated 22Rv1 or 22Rv1-KO cells. The flow cytometry analyses were performed for the same time points as the Luminex assays, i.e., at day 0, day 6 (after two simulations with 22Rv1/22Rv1-KO cells), and day 13 (3 days after the fourth stimulation). 

**Figure 7:**
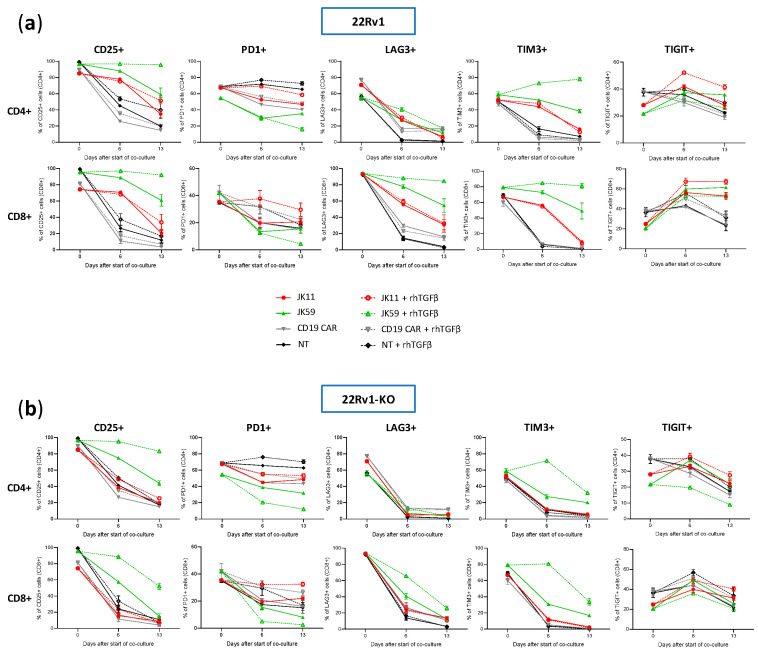
Analysis of STEAP1 and SwR-STEAP1 CAR T cell phenotyping after repeated in vitro stimulation of STEAP1^+^ tumor cells. Long-term co-culture was achieved by stimulating every 3 to 4 days the JK11, JK59, and CD19 CAR T cells and NT T cells, with irradiated 22Rv1 and 22Rv1-KO target cells at an E:T ratio of 1:1 in the absence or presence of 10 ng/mL rhTGFβ. Before the start of the co-culture (day 0), at day 6, and day 13, CAR T cell phenotyping was assessed with flow cytometry. (**a**,**b**) The CD4^+^ and CD8^+^ T cell populations co-cultured with 22Rv1 target cells (**a**) or with 22Rv1-KO target cells (**b**) were assessed for surface expression of the activation marker CD25 and the checkpoint receptors PD1, LAG3, TIM3, and TIGIT. Background staining for each marker was identified using Fluorescence Minus One controls (see Figure S7a–c for gating strategy). Data represent the mean values ± SEM from two healthy donors. Each co-culture was performed in duplicate, and these were kept separate throughout the assay.

The expression of checkpoint molecules/activation markers CD25, PD-1, LAG3, TIGIT, and TIM3 were measured using the gating strategy shown in Figure S7a–c. At day 0, the T cells generally expressed high levels of these molecules, suggesting an activated T cell phenotype (Figure 7a,b). After repeated stimulations, the JK11 CAR T cells expressed less CD25, LAG3, TIM3, and TIGIT, whereas their PD-1 level was relatively stable. In contrast, the JK59 CAR T cells supplemented with TGFβ gradually expressed less PD-1 but remained mostly CD25^+^, and also retained a considerably higher expression of LAG3 (in CD8^+^ T cells) and TIM3 (Figure 7a). To a lesser extent, these differences also applied to JK59 CAR T cultures without TGFβ.

**Figure 8:**
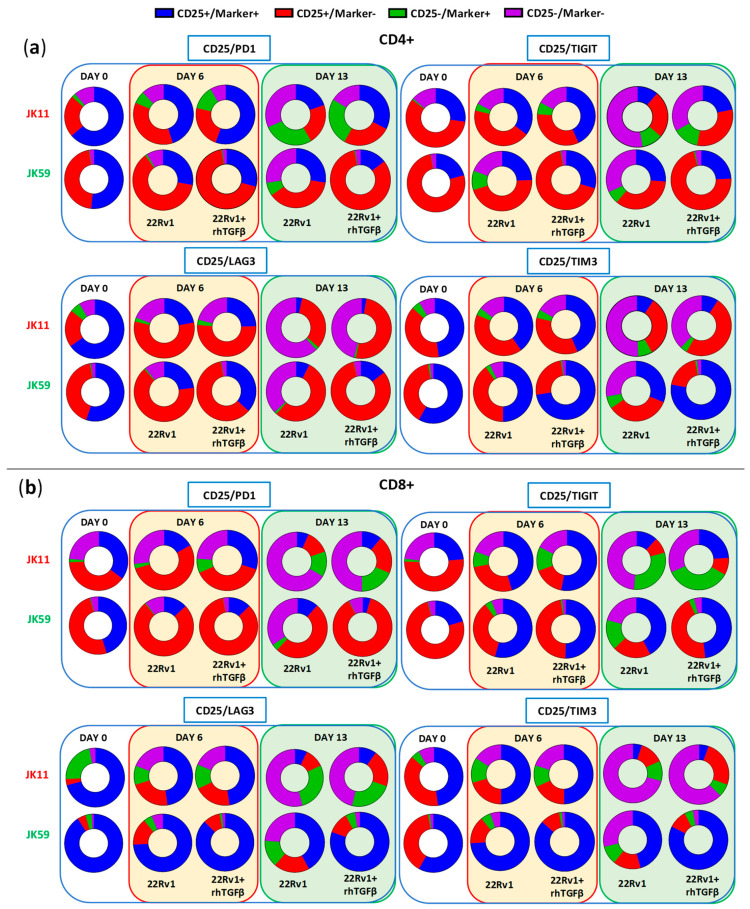
SwR-STEAP1 CAR T cells show a higher polyfunctionality after repeated in vitro stimulation of tumor cells. ‘Parts of Whole’ graphs showing the co-expression of the activation maker CD25 with the checkpoint receptors PD1, LAG3, TIM3, and TIGIT on CD4^+^ (**a**) and CD8^+^ (**b**) JK11 and JK59 CAR T cells. Samples were collected before the start of long-term co-culture assay (day 0) and at days 6 and 13 of co-culture. T cells were co-cultured with 22Rv1 cells or with 22Rv1 target cells in the presence of rhTGFβ (22Rv1 + rhTGFβ). Co-cultures were performed at an E:T ratio of 1:1 (see also Figure 7 and Figure S8). Average expression values from the two healthy donors of the long-term co-culture assay were used. Each co-culture from each donor was performed in duplicate.

Figure 8 shows the proportion of single cells co-expressing the activation marker CD25 with checkpoint molecules PD-1, LAG3, TIGIT, or TIM3. T cells expressing CD25, but not PD-1, may represent an activated, non-exhausted phenotype. The JK59 CAR T cells had a higher proportion of this phenotype, both for CD4^+^ and CD8^+^, compared to the JK11 CAR T cell population. The difference became larger during culture with TGFβ. At day 13, 82%/88% of CD4^+^/CD8^+^ JK59 CAR T cells cultured with 22Rv1-TGFβ were CD25^+^PD-1^−^, whereas only 25%/19% of the CD4^+^/CD8^+^ JK11 CAR T cells had this phenotype. The proportions of single cells co-expressing CD25 with LAG3, TIGIT, or TIM3 were also considerably higher in the JK59 CAR T cells. After 13 days of culture with 22Rv1-TGFβ, 77%/82% of CD4^+^/CD8^+^ JK59 CAR T cells were CD25^+^TIM3^+^, and 24%/48% of CD4^+^/CD8^+^ T cells were CD25^+^/TIGIT^+^. LAG3 was mainly expressed in CD8^+^ T cells, where 80% were CD25^+^LAG3^+^. The expression of checkpoint molecules LAG3, TIGIT, and TIM3 decreased over the 13 days in JK59 CAR T cells cultured with 22Rv1-KO plus TGFβ and, thus, appeared STEAP1-dependent (Figure 7a,b). Figure S8 shows the proportion of single cells co-expressing CD25 with PD-1, LAG3, TIGIT, or TIM3 when co-cultured with 22Rv1-KO cells +/− rhTGFβ. This proportion was substantially lower at day 13 for JK59 CAR T cells cultured with 22Rv1-KO + rhTGFβ, compared to wild-type 22Rv1 + rhTGFβ.

The T cells were, furthermore, classified into four differentiation subsets: naive, effector memory (EM), central memory (CM), or T effector memory re-expressing CD45RA (terminal differentiated; TEMRA). At day 0, ~80% of the JK11 and JK59 CAR T cells were CM cells (Figure S9). Upon stimulation with STEAP1^+^ 22Rv1 cells, the proportion of EM cells increased to ~80%, and the CM proportion decreased correspondingly (Figure S9a). CAR T differentiation from CM to EM was more prominent in CAR T cells cultured with STEAP1^+^ 22RV1 cells, compared to STEAP1 KO controls (Figure S9b), and thus appeared to be partially STEAP1-dependent. The proportion of TEMRA was < 2% and < 13% in CD4^+^ and CD8^+^ T cells, respectively, throughout the 14 days for all CAR T variants co-cultured with STEAP1^+^ 22Rv1 cells. In JK59 CAR T cells cultured with 22Rv1-KO + rhTGFβ, the naive and TEMRA proportions increased over the 14 days (Figure S9b).

The results indicated that most JK59 CAR T cells had an EM phenotype after four stimulations with 22Rv1 + rhTGFβ and expressed multiple activation markers but not PD-1. This observation suggested that the T cells were activated but not exhausted, and was consistent with the data described above from functional T cells assays (Caspase3, flow cytometry cytokines, Luminex, cell counts).

## 4. Discussion

The main purpose of this study was to develop a construct conferring tumor-targeting T cells with improved functionality in a TGFβ-rich environment. The results suggest that the described switch receptor (SwR) fulfills this purpose for the STEAP1 CAR T cells, both as measured by T cell proliferation, cytokine response, cytotoxicity, and phenotypic development upon repeated/persisting antigen exposure. We cloned a TGFβ-SwR, in which the extracellular domain of the TGFβ receptor is fused to intracellular domains from the IL-2/15 receptor. The SwR was cloned in tandem with our STEAP1 CARs JK11 (CD8 spacer) and JK15 (IgG1 spacer) into single constructs, which gave co-expression of the SwR and CAR in primary T cells after retroviral transduction. The two SwR-CAR constructs (JK59 and JK69) were expressed at similar levels and conferred both CD4^+^ and CD8^+^ T cells with STEAP1-specific anti-tumor activity. TGFβ inhibited the ability of T cells expressing only the CAR to proliferate and decreased their capability to kill cancer cells but increased the expansion of SwR-CAR T cells. In TGFβ-rich environments, the SwR-CAR T cells had a substantially more potent response against STEAP1^+^ target cells, compared to the JK11 and JK15 CAR T cells, as measured by cytotoxicity and cytokine response (flow cytometry, Luminex). Long-term assays, where the T cells were stimulated four times with target cells plus TGFβ, showed that the JK59 CAR T cells proliferated and evolved into effector memory cells, characterized by the expression of multiple activation markers, combined with low PD-1 expression. Upon a fifth re-challenge with target cells, the JK59 CAR T cells showed retained and STEAP1-specific functionality.

The expression of large constructs is generally inferior to the expression of smaller ones, and this represents a hurdle for the development of add-on constructs for CAR T cell therapy. In freshly transduced T cells, we did observe that the expression of the SwR-CAR constructs (JK59, JK69) was substantially lower than for JK11 and JK15. However, the SwR-CAR T response in cytotoxicity and cytokine assays was still similar. After freezing/thawing and 2 days of re-activation with anti-CD3/CD28 antibodies, the JK59 and JK69 CAR expression increased and reached the same level as JK11 and JK15 (80–90%). The explanation for this is not clear, but the integration of retroviral constructs into cellular DNA depends on proliferation, and T cells expressing the SwR-CAR construct would be expected to be more viable after freezing/thawing and more proliferative. T cell products for clinical application are usually frozen/thawed. The data suggest that the SwR is sufficiently well co-expressed with either of the STEAP1 CARs, but this would have to be validated in a clinical manufacturing protocol. 

The anti-tumor response tended to be stronger for the SwR CAR T cells (JK59, JK69) than for T cells with a stand-alone CAR (JK11, JK15), even without supplemented TGFβ. This may be explained by a ligand-independent stimulatory effect of the ICD expressed in the SwR, or stimulation from small amounts of TGFβ secreted by 22Rv1 cells, or by the CAR T cells themselves. We found that supplemented TGFβ had a suppressive effect on the function of STEAP1 CAR T cells, as has been reported in many T cell models. The suppressive effect was observed for T cell proliferation, cytotoxicity, and cytokine response. The STEAP1 CAR T cells did, however, retain a substantial level of anti-tumor activity. This may reflect that CARs generally provide powerful stimulatory signals, compared to natural TCRs, and that the JK11 construct is highly expressed and includes 4-1BB.

In the present study, we used a double negative, non-signaling TGFβ-receptor (Dnm) as a reference, similar to the one that has been tested in patients with castration-resistant metastatic prostate cancer [18]. The expression of the Dnm-CAR construct was higher than for the JK59 and JK69 SwR constructs in freshly transduced T cells, while the expression levels were similar after freezing/thawing and 2 days of T cell activation. In a TGFβ-rich environment, CAR T cells transduced with the SwR proliferated substantially more than the Dnm CAR T cells. This finding indicates that the SwR is functional and confers a stimulatory signal, and does not merely nullify TGFβ-mediated suppression.

The cytokine profile was Th1-weighted, with high levels of TNFα and IFNγ and low levels of IL-4 and IL-10. We still detected substantial levels of the Th2-associated cytokine IL-13. A mixed Th1/Th2 profile is in line with observations we have previously made for T cells transduced with tumor-targeting TCRs [38,39] or the JK11 STEAP1 CAR [22] and in studies of cancer vaccine responses [40,41], where a strict Th1/Th2 dichotomy did not apply even in monoclonal T cell cultures [42]. In the present study, we further found that the SwR CAR T cells secreted multiple chemokines and other pro-inflammatory cytokines. This may be favorable and indicate a capacity to transform the tumor microenvironment. However, the observed cytokine profiles should be interpreted with caution, as it is difficult to predict the in vivo cytokine levels based on in vitro measurements. It is still of interest that the cytokine response of JK11 CAR T cells, but not SwR CAR T cells, appeared to be attenuated by TGFβ.

As mentioned above, IL-2 and IL-15 have different effects on T cells [19,20], even if the intracellular signaling pathways downstream of IL-2/IL-15 stimulation are mostly similar. The different effects of IL-2 and IL-15 underline the point that the effect of TGFβ-stimulation of the SwR is hard to predict theoretically and likely to be influenced by the binding properties of TGFβ. We investigated both the properties of the initial SwR CAR T response and the effect of repeated stimulations over time. The results indicate that, in a TGFβ-rich environment, the SwR confers CAR T cells enhanced cytotoxic activity and cytokine response, as well as increased proliferation and survival. It is of particular interest that the JK59 SwR CAR T cells developed a phenotype associated with activation but not AICD or exhaustion. This could be important for the use of the SwR in a clinical setting. It should be noted, though, that this may depend on multiple factors that will differ between the in vitro assays and the patient setting. There is a need to investigate the effect of the SwR in relevant in vivo models. It is important to determine if the favorable effect of the SwR on CAR T cell potency, expansion, and survival applies in vivo and how it is affected by physiological concentrations of TGFβ, which will vary between the tumor microenvironment, peripheral blood, and other compartments. 

TGFβ has multiple and complex effects on the tumor microenvironment. Therapies targeting TGFβ have not so far shown convincing clinical efficacy [43]. This may be because TGFβ both promotes and counters cancer development. It has been described that TGFβ inhibits tumor cell proliferation and induces apoptosis of cancer cells while also suppressing the anti-cancer immune response. Further, the systemic administration of agents targeting TGFβ may give side effects that make it difficult to reach sufficient dosing for obtaining the desired effect on the local immune response. In this respect, the TGFβ SwR approach described herein appears attractive, as the TGFβ-axis would only change in the injected T cells. This means that TGFβ-effects in normal host cells can be retained, that T cells in normal tissues should not be stimulated, and that any anti-cancer effects of TGFβ may be preserved. The approach may be applicable to both CAR T cells and other forms of adoptive cell therapy, including therapy with NK cells, tumor-infiltrating lymphocytes, or T cells redirected with tumor-targeting T cell receptors. 

A possible safety concern would be an enhanced activity of the SwR-expressing T cells in any environment with high TGFβ concentrations, not only in the tumor. In the assays reported above, we observed that the JK59 CAR T cells survived for 14 days in STEAP1 KO cultures supplemented with only TGFβ. However, the increase in T cell numbers was STEAP1-dependent, as was the cytotoxic activity and cytokine response. These observations suggest that the JK59 CAR T cells have retained STEAP1-specificity. For improved safety in a first-in-man trial, transient mRNA-based expression of the SwR may be considered [38,44,45].

In conclusion, we have cloned and expressed an SwR fusing the TGFβ receptor I and II exodomains with the endodomain of the IL-2/IL-15 receptors β and γ chains. The TGFβ SwR was co-expressed with STEAP1 CARs and conferred upon the CAR T cells an improved in vitro functionality in TGFβ-rich environments, as measured by T cell proliferation/survival, cytokine response, and cytotoxicity. Data from two-week assays indicated that the SwR CAR T cells developed an activated, effector memory phenotype with retained functional potency upon repeated and persisting antigen exposure. Further studies are required to investigate the in vivo functionality of the SwR CAR T cells. The SwR may be used as an add-on construct for CAR T cells and may also be applicable to other forms of adoptive cell therapy.

## Data Availability

Data supporting the reported results can be obtained from the corresponding author.

## Supplementary Materials

The following supporting information can be downloaded at: https://www.mdpi.com/article/10.3390/biomedicines11020459/s1, Figure S1: Dnm and SwR-STEAP1 CAR T cells phenotyping, viability and recovery yield; Figure S2: STEAP1-IgG and SwR-STEAP1-IgG CAR design and expression; Figure S3: STEAP1 expression and TGFβ secretion in prostate cancer cell lines; Figure S4: Gating strategy for TNFα and IFNγ cytokines production and apoptotic target cells; Figure S5: TGFβ enhances the proliferation and functionality of SwR-STEAP1 CAR T after repeated in vitro stimulation of STEAP1+ C4-2B tumor cells; Figure S6: Cytokines and chemokines profile of STEAP1 and SwR-STEAP1 CAR T cells co-cultured with 22Rv1-KO cells in the presence of rhTGFβ; Figure S7: Gating strategy of the CAR T cell phenotyping from the long-term co-culture assay; Figure S8: Analysis of STEAP1 and SwR-STEAP1 CAR T cells polyfunctionality during the long-term co-culture assay with STEAP1 knockout target cells; Figure S9: Different T cell subsets from the long-term co-culture assay.
