# Supplementary material for: Development of a TGFβ—IL-2/15 Switch Receptor for Use in Adoptive Cell Therapy
Source: Biomedicines. 2023 Feb 4;11(2):459. doi: 10.3390/biomedicines11020459 (PMC9953633; doi:10.3390/biomedicines11020459)
Supplement: Supplementary file 1 [file biomedicines-11-00459-s001.zip › biomedicines-2109354-supplementary.pdf]

Figure S1

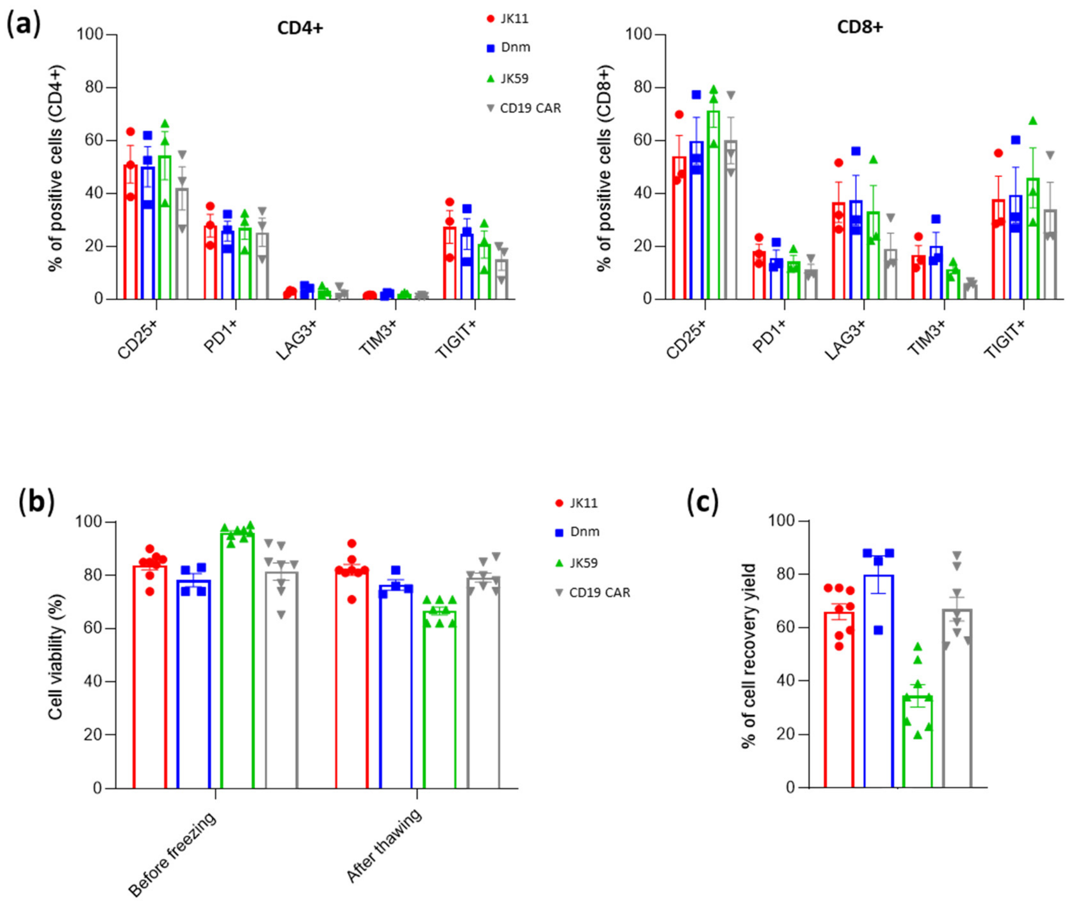

Figure S1. Dnm and SwR-STEAP1 CAR T cells phenotyping, viability and recovery yield. (a) PBMC isolated from three healthy donors were stimulated for 2 days with anti-CD3/CD28 antibodies and rhIL-2 before retroviral transduction with the JK11, Dnm, JK59 and CD19 CARs. The cells were expanded an additional 9 days before phenotyping by flow cytometry. Graphs show the proportion of the activation marker CD25, and the checkpoint receptors PD1, LAG3, TIM3 and TIGIT expression on $CD4^+$ (left) and $CD8^+$ (right) T cells. Background staining for each marker was identified using Fluorescence Minus One controls. The gating strategy used was similar to the one used in figure 7 (see Figure S7a-c for gating strategy). Data represents the mean values ± SEM from three healthy donors (duplicate samples from each donor). **(b)** A graph showing the CAR T cell viability before cryopreservation and after thawing and re-activation for 2 days with anti-CD3/CD28 antibodies and rhIL-2. **(c)** A graph showing the recovery yield of JK11, Dnm, JK59 and CD19 CAR T cells. T cell number and viability were determined before cryopreservation (6 days after transduction), and after thawing and re-activation for 2 days with anti-CD3/CD28 antibodies and rhIL-2. Cell viability and cell counts were measured by labelling the T cells with trypan blue, and counted using a Countess™ Automated Cell Counter. In **(b)** and **(c)** Data represents the mean values ± SEM from four to eight healthy donors.

Figure S2

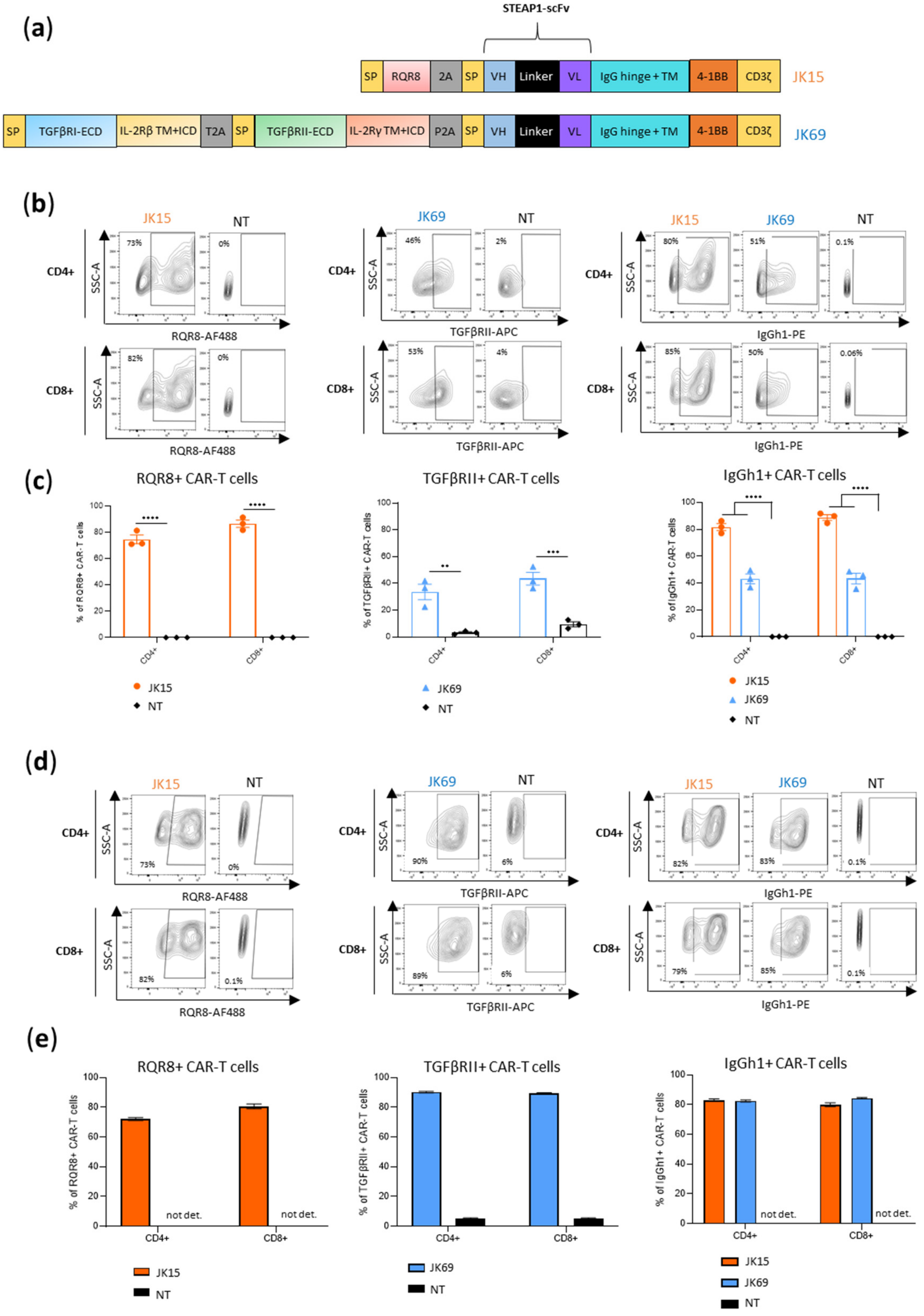

Figure S2. STEAP1-IgG and SwR-STEAP1-IgG CAR design and expression. (**a**) Schematic representation of the STEAP1-IgG (JK15) CAR and the TGFβ-switch receptor (SwR)-IgG (JK69) CAR with the extracellular domain (ECD) of the TGFβRI and TGFβRII subunits fused to the transmembrane domain (TM) and intracellular domain (ICD) of the IL-2/IL-15 receptor β and γ chains, respectively (IL-2Rβ and IL-2Rγ). Both CARs contain the human IgG1 (IgGh1) hinge and TM domain. (**b**) and (**c**) Flow analysis of the CAR expression in primary human T cells stained with CD3, CD4 and CD8, and with the antibody recognizing the RQR8 sequence of JK15 CAR (left panel),

with the TGFβRII antibody recognizing JK69 CAR (middle panel), or with the antibody recognizing the IgGh1 hinge (right panel) in the JK15 and JK69 CARs. Non-transduced (NT) T cells were used as controls. Data represents the mean values ± SEM of three different T cell transductions from three different healthy donors. Each transduction was done in duplicate. (**d**) and (**e**) Flow analysis of the CAR expression in human T cells after CAR T cells freezing/thawing and activation with anti-CD3/CD28 antibodies for 2 days. (**d**) Contour plots of JK15 and JK69 CAR expression. (**e**) Graphs representing the expression of JK15 and JK69 CARs after freezing/thawing and activation from one healthy donor. Data represents the mean values ± SEM. Data were analyzed by one-way ANOVA with Tukey's multiple comparisons test. **p<0.01; ***p<0.001; ****p<0.0001. not det.: = not detected.

Figure S3

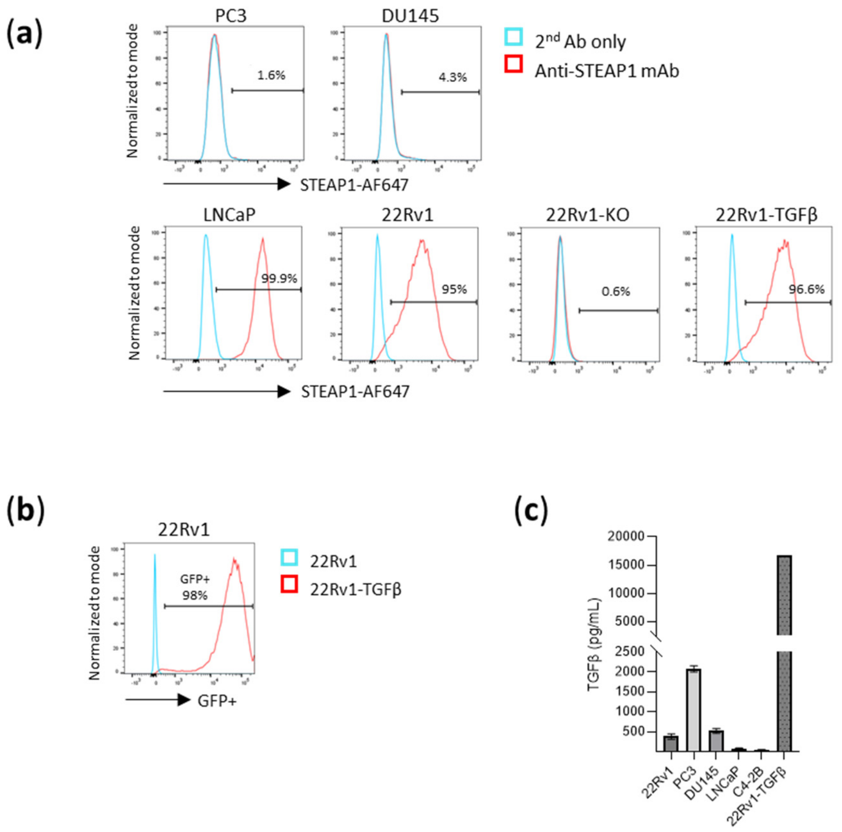

Figure S3. STEAP1 expression and TGFβ secretion in prostate cancer cell lines. (**a**) Flow cytometry analysis of STEAP1 expression in PC3, DU145, LNCaP and 22Rv1 prostate cancer cell lines, as well as in 22Rv1-KO cells, and 22Rv1 cells overexpressing TGFβ (22Rv1-TGFβ). Cells were stained with an anti-STEAP1 mAb followed by an AF647 secondary antibody (red histogram), or with the secondary antibody only (2nd Ab only; blue histogram). (**b**) ELISA determination of secreted TGFβ in cell culture supernatant, from 22Rv1, LNCaP, C4-2B, PC3 and DU145 prostate cancer cell lines, as well as the 22Rv1 cell line overexpressing TGFβ (22Rv1-TGFβ). Data represents the mean values ± SEM of 3 independent experiments, with each experiment performed in duplicate. (**c**) TGFβ is co-expressed with the marker gene EGFP. The TGFβ level can then be measured by the level of GFP by flow cytometry. Representative histogram of the expression level of GFP in 22Rv1 (blue) and 22Rv1-TGFβ (red) cell lines, measured by flow cytometry.

Figure S4

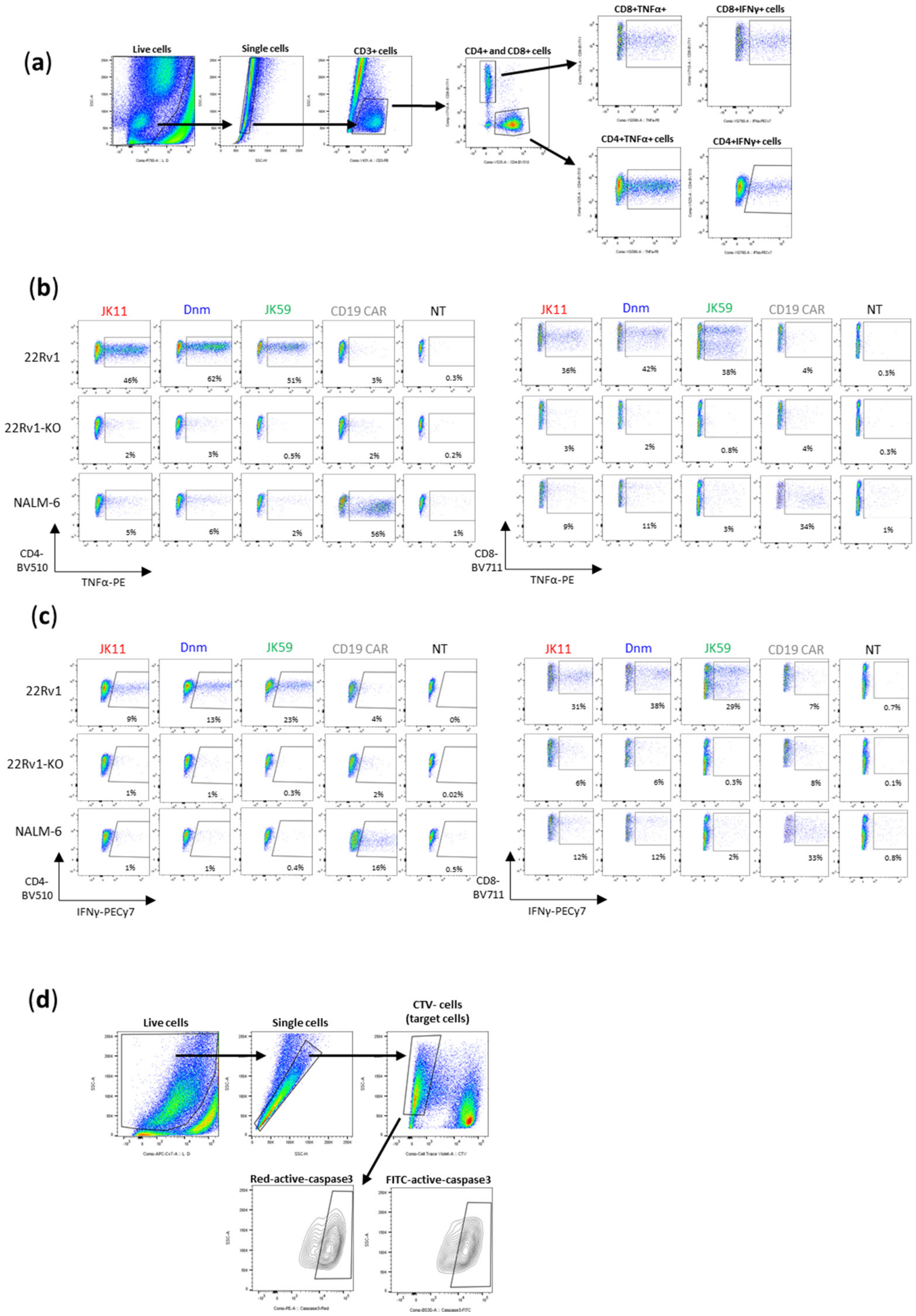

Figure S4. Gating strategy for TNFα and IFNγ cytokines production and apoptotic target cells. (**a**) Gating strategy of TNFα and IFNγ production in effector cells. Dead cells were excluded using the Fixable Dye eFluor™ 780. Doublets were excluded, and effector T cells were selected by CD3, CD4 and CD8 staining. From CD4⁺ and CD8⁺ population, the positive populations of TNFα and IFNγ were gated. **(b)** Representative dot plots of the production of TNFα in the CD4⁺ (left panel) and CD8⁺ (right panel) T cell groups, or the NT control group. **(c)** Representative dot plots of the production of IFNγ in the CD4⁺ (left panel) and CD8⁺ (right panel) T cell groups, or the NT control group. (**d**) Gating strategy of apoptotic target cells. Dead cells were excluded with the Fixable Dye eFluor™ 780. Doublets were excluded, effector CAR T cells were excluded by the CellTrace™ Violet (CTV) staining, and apoptotic cells were stained with FITC- or Red-DEVD-FMK dye.

Figure S5

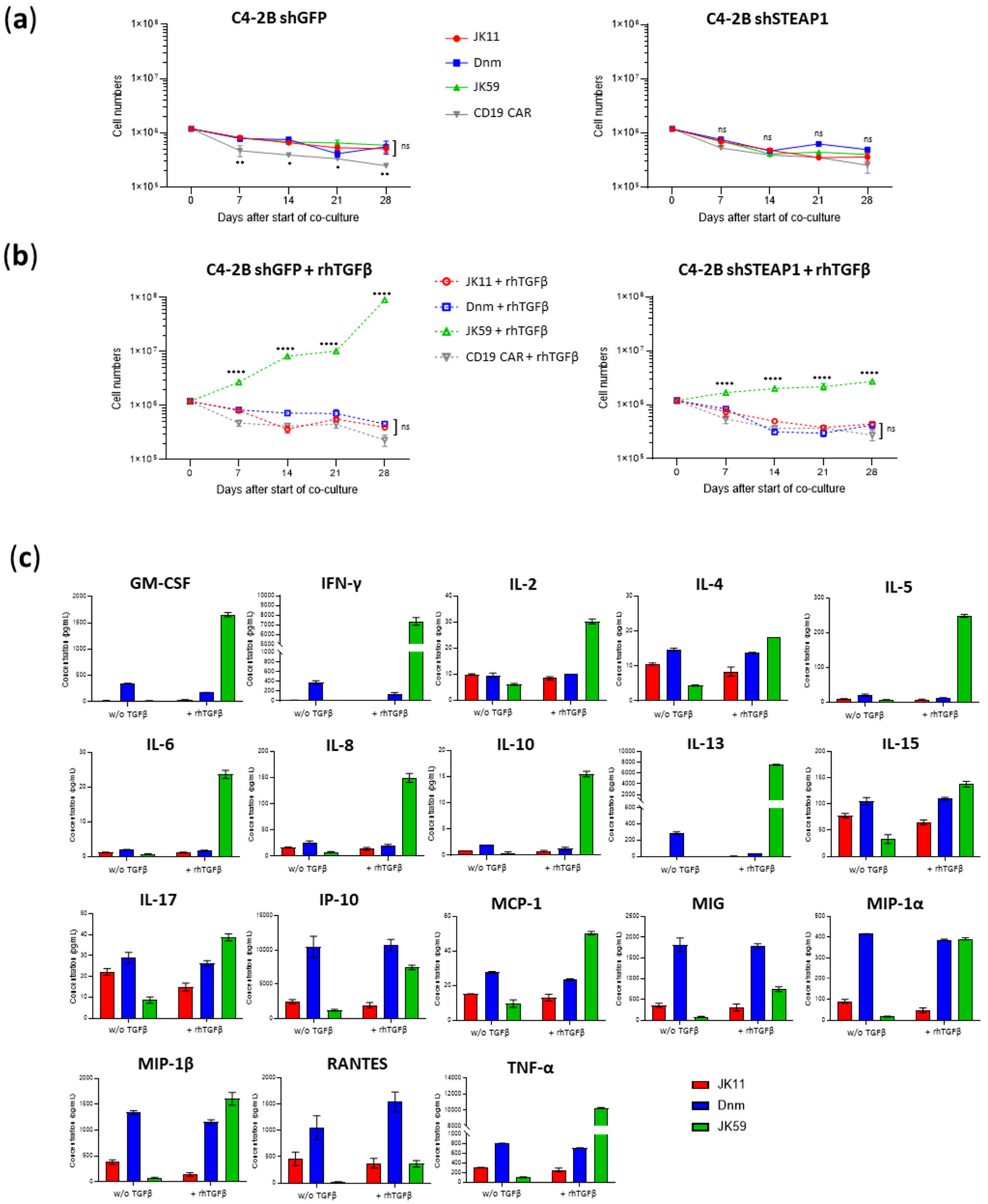

Figure S5. TGFβ enhances the proliferation and functionality of SwR-STEAP1 CAR T after repeated in vitro stimulation of STEAP1⁺ C4-2B tumor cells. Freshly transduced JK11, Dnm, JK59 and CD19 CAR T cells were co-cultured with irradiated (20 Gy) C4-2B shGFP and C4-2B shSTEAP1 target cells, in absence or presence of 5 ng/mL rhTGFβ, at an E:T ratio of 1:1 for 7 days. Every 7 days, the T cell number was measured, and the same T cells were co-cultured again on freshly irradiated target cells for 7 more days until day 28. (a) A graph representing the number of T cells following co-culture with C4-2B shGFP (left panel) or C4-2B shSTEAP1 (right panel) target cells at days 0, 7, 14, 21 and 28. (b) A graph representing the number of T cells after co-culture with C4-2B shGFP (left panel) or C4-2B shSTEAP1 (right panel) target cells at days 0, 7, 14, 21 and 28, in presence of 5 ng/mL rhTGFβ. Data in (a) and (b) represents the mean values ± SEM of one healthy donor. Each co-culture was performed in duplicate, and each duplicate pair was kept separate

until the end of the experiment. Data were determined by two-way ANOVA with Tukey's multiple comparisons test. *p<0.05; **p<0.01; ****p<0.0001; ns = not significant. (c) Luminex cytokine analyses were performed using T cells supernatants isolated at day 18 from JK11, Dnm, and JK59 CAR T cells, co-cultured with C4-2B shGFP in presence or absence (w/o) of 5 ng/mL rhTGFβ. Data represents the mean values ± SEM of duplicate from one healthy donor.

Figure S6

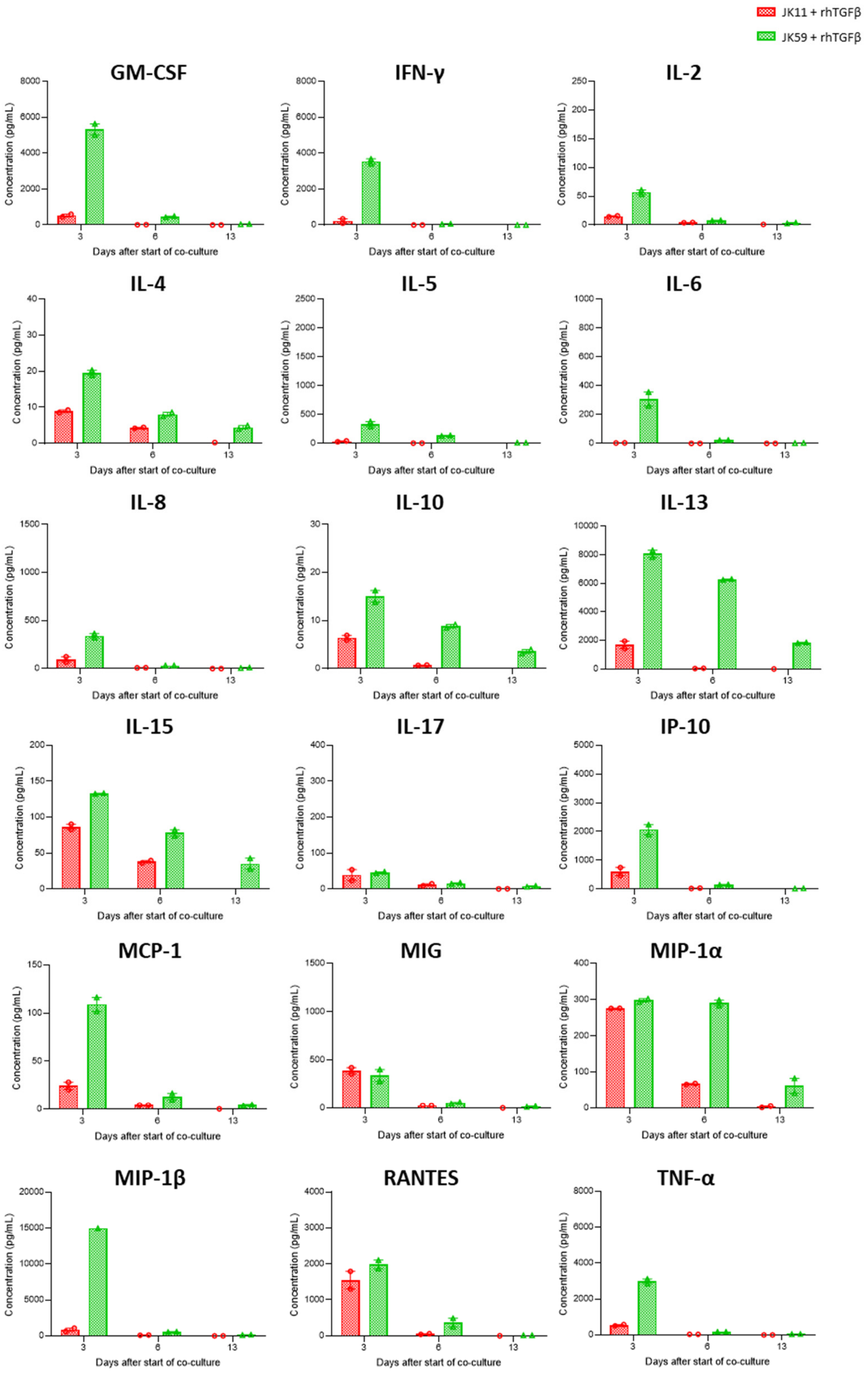

Figure S6. Cytokines and chemokines profile of STEAP1 and SwR-STEAP1 CAR T cells co-cultured with 22Rv1-KO cells in the presence of rhTGFβ. Luminex cytokine analyses were performed using T cell supernatants isolated at days 3, 6 and 13 from JK11 and JK59 CAR T cells co-cultured with 22Rv1-KO target cells in presence of 10 ng/mL rhTGFβ, as shown in Figure 5a and 5b. Data represents the mean values ± SEM of two healthy donors. Each co-culture from each donor were done in duplicate and each duplicate were kept separate throughout the experiment.

Figure S7

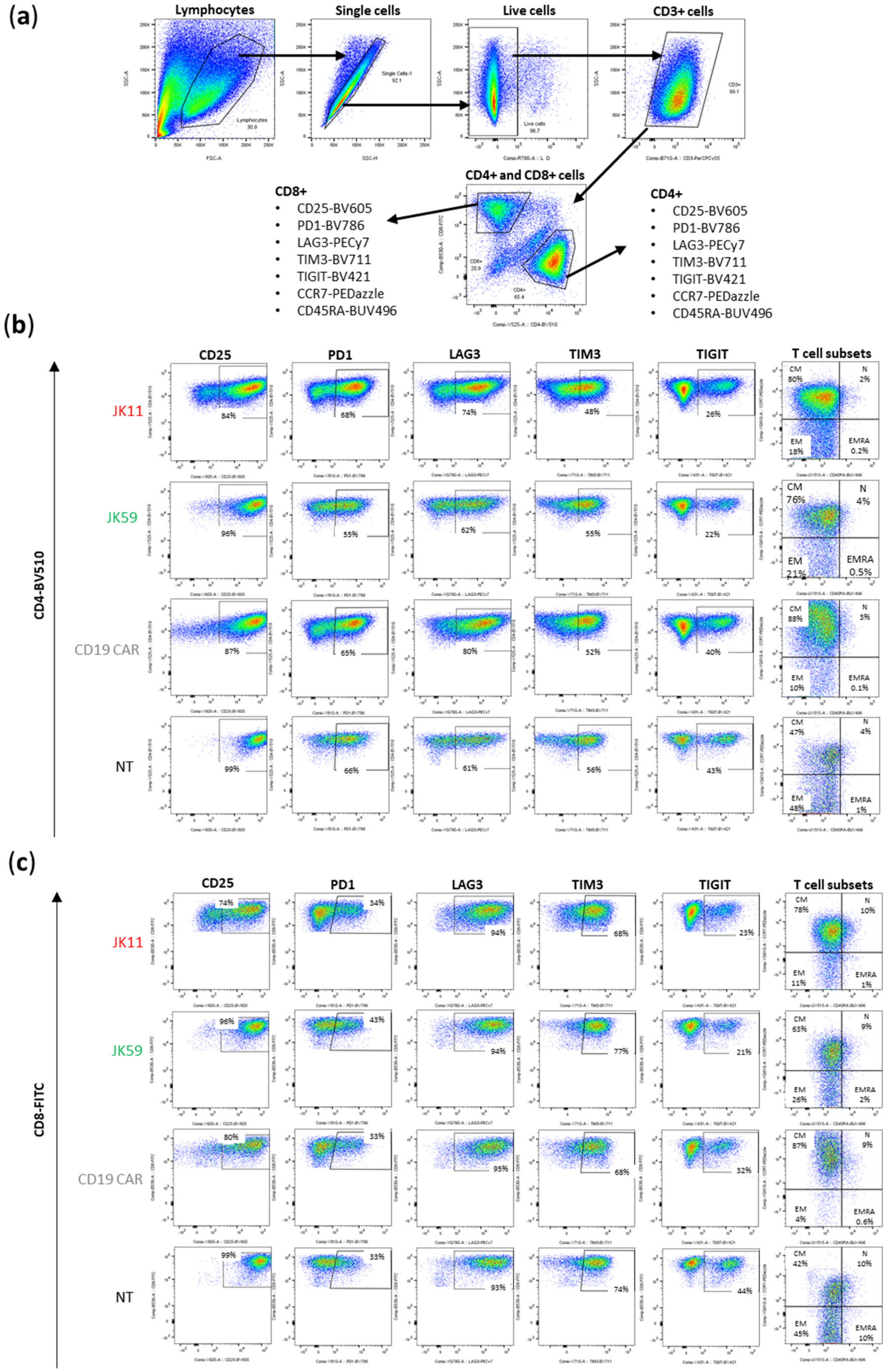
(a)
Lymphocytes
Single cells
Live cells
CD3+ cells
CD4+ and CD8+ cells
CD8+
CD25-BV605
PD1-BV786
LAG3-PECy7
TIM3-BV711
TIGIT-BV421
CCR7-PEDazzle
CD45RA-BUV496
CD4+
CD25-BV605
PD1-BV786
LAG3-PECy7
TIM3-BV711
TIGIT-BV421
CCR7-PEDazzle
CD45RA-BUV496
(b)
CD25
PD1
LAG3
TIM3
TIGIT
T cell subsets
CD4-BV510
JK11
84%
68%
74%
48%
26%
CM 80%
N 2%
EM 18%
EMRA 0.2%
JK59
96%
55%
62%
55%
22%
CM 76%
N 4%
EM 21%
EMRA 0.5%
CD19 CAR
87%
65%
80%
52%
40%
CM 88%
N 3%
EM 10%
EMRA 0.1%
NT
99%
66%
61%
56%
43%
CM 47%
N 4%
EM 48%
EMRA 1%
(c)
CD25
PD1
LAG3
TIM3
TIGIT
T cell subsets
CD8-FITC
JK11
74%
34%
94%
68%
23%
CM 78%
N 10%
EM 11%
EMRA 1%
JK59
96%
43%
94%
77%
21%
CM 63%
N 9%
EM 26%
EMRA 2%
CD19 CAR
80%
33%
95%
68%
32%
CM 87%
N 9%
EM 4%
EMRA 0.6%
NT
99%
33%
93%
74%
44%
CM 42%
N 10%
EM 45%
EMRA 10%

Figure S7. Gating strategy of the CAR T cell phenotyping from the long-term co-culture assay. (**a**) Gating strategy of JK11, JK59 and CD19 CAR T cells, and NT T cells co-cultured with 22Rv1 and 22Rv1-KO target cells in the absence or presence of 10 ng/mL rhTGFβ, at an E:T ratio of 1:1 at days 0, 3, 6, 10 and 13. Representative dot plot of T cells (from day 0) gated on SSC-A and FSC-A, followed by exclusion of doublets. Dead cells were excluded with the Fixable Dye eFluor™ 780 and T cells were gated on $CD3^+$, $CD4^+$ and $CD8^+$ populations. The $CD4^+$ and $CD8^+$ T cell populations were assessed for surface expression of the activation marker CD25 and the checkpoint receptors PD1, LAG3, TIM3 and TIGIT, as well as the expression of CCR7 and CD45RA to differentiate the different T cell subsets (Naïve; EM: effector memory; CM: central memory; TEMRA: T effector memory re-expressing CD45RA). (**b**) and (**c**) Representative dot plots (at day 0, from the long-term co-culture assay) of the expression of CD25, PD1, LAG3, TIM3, TIGIT, CCR7 and CD45RA from the total $CD4^+$ (**b**) and $CD8^+$ (**c**) T cell population. Background staining for each marker was defined using Fluorescence Minus One controls. The same gating strategy was used for CAR T cell phenotyping at days 6 and 13 of the long-term co-culture assay.

Figure S8

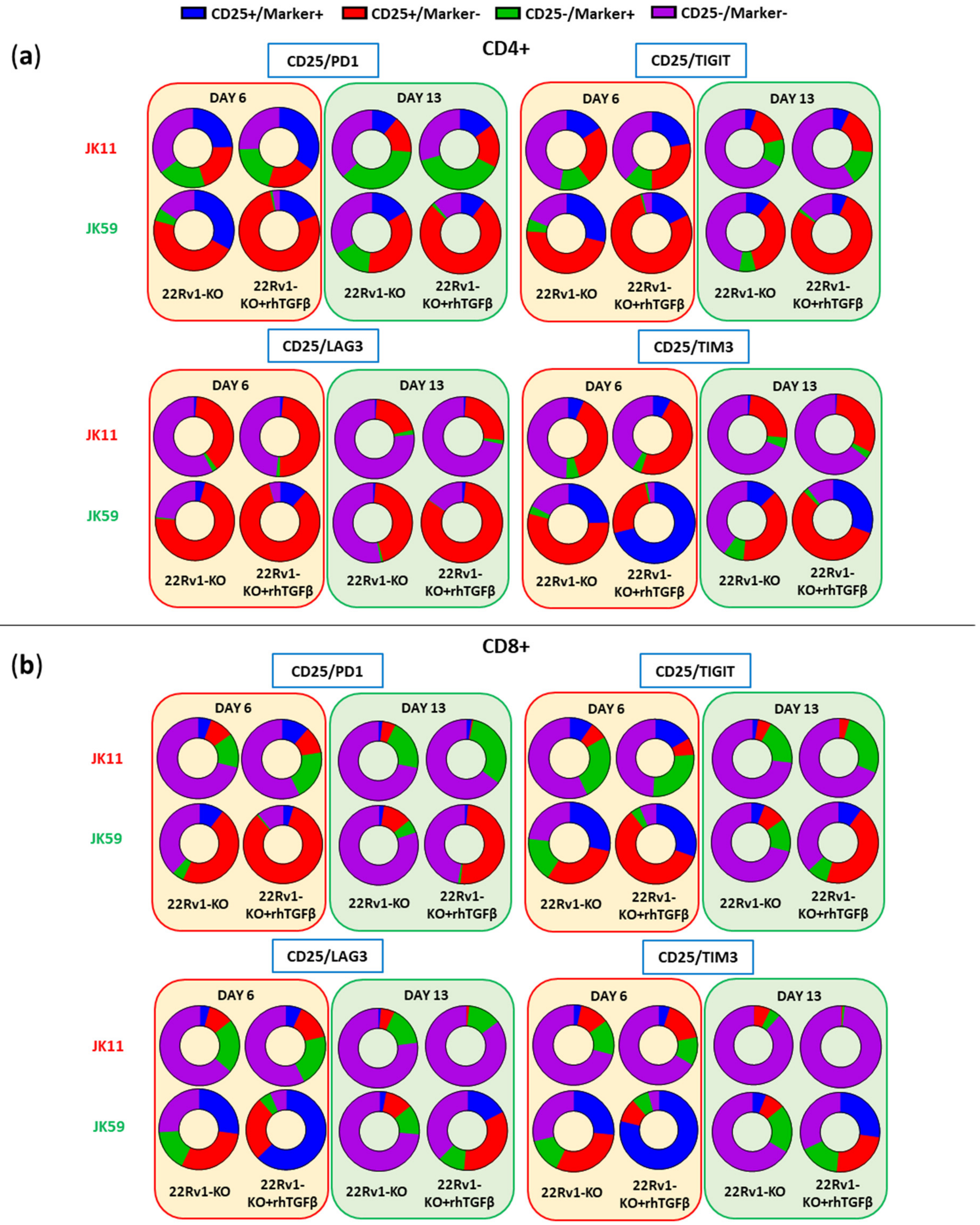

Figure S8. Analysis of STEAP1 and SwR-STEAP1 CAR T cells polyfunctionality during the long-term co-culture assay with STEAP1 knockout target cells. 'Parts of Whole' graphs showing the co-expression of the activation maker CD25 with the checkpoint receptors PD1, LAG3, TIM3 and TIGIT on CD4+ (a) and CD8+ (b) JK11 and JK59 CAR T cells co-cultured with 22Rv1-KO and 22Rv1-KO target cells in presence of rhTGFβ (22Rv1-KO + rhTGFβ) at an E:T ratio of 1:1 at days 6 and 13 (see also Figure 7 and 8). Average expression values from the two healthy donors of the long-term co-culture assay were used. Each co-culture from each donor were done in duplicate.

Figure S9

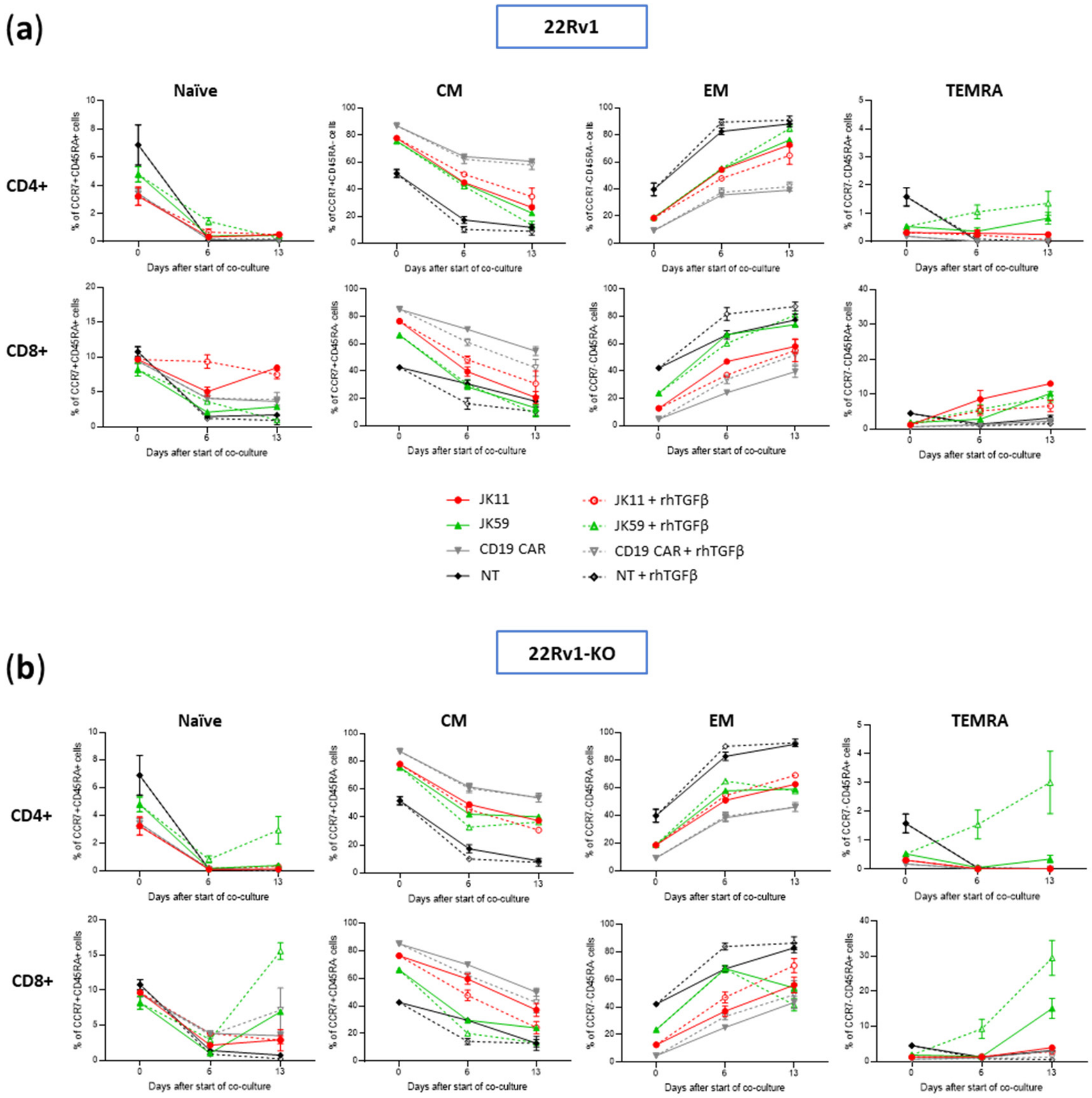

Figure S9. Different T cell subsets from the long-term co-culture assay. Long-term co-culture was achieved by stimulating every 3 to 4 days the JK11, JK59 and CD19 CAR T cells, and NT T cells, with irradiated 22Rv1 and 22Rv1-KO target cells at an E:T ratio of 1:1, in the absence or presence of 10 ng/mL rhTGFβ. Before the start of the co-culture (day 0), at day 6, and at day 13, the T cell subsets were assessed by flow cytometry using the CCR7 and CD45RA markers (Naïve; EM: effector memory; CM: central memory; TEMRA: T effector memory re-expressing CD45RA). (**a**) The $CD4^+$ and $CD8^+$ T cell populations co-cultured with 22Rv1 target cells. (**b**) The $CD4^+$ and $CD8^+$ T cell populations co-cultured with 22Rv1-KO cells. Background staining for each marker was defined using FMOs (see also Figure S7 for gating strategy). Data represents the mean values ± SEM of two healthy donors. Each co-culture was done in duplicate, and these were kept separate throughout the assay.
